# Testosterone promotes either dominance or submissiveness in the Ultimatum Game depending on players’ social rank

**DOI:** 10.1038/s41598-017-05603-7

**Published:** 2017-07-13

**Authors:** Yukako Inoue, Taiki Takahashi, Robert P. Burriss, Sakura Arai, Toshikazu Hasegawa, Toshio Yamagishi, Toko Kiyonari

**Affiliations:** 1 Department of Cognitive and Behavioral Science, Graduate School of Arts and Sciences, The University of Tokyo, Tokyo, 153-8902 Japan; 2 Graduate School of Letters, Hokkaido University, Sapporo, 060-0810 Japan; 3 Faculty of Psychology, Basel University, Basel, 4055 Switzerland; 4 Department of Psychological and Brain Sciences, University of California, Santa Barbara, California, 93106 USA; 5 Brain Science Institute, Tamagawa University, Machida, 194-8610 Japan; 6 Graduate School of International Corporate Strategy, Hitotsubashi University, Tokyo, 101-8439 Japan; 7 School of Social Informatics, Aoyama Gakuin University, Sagamihara, 252-5258 Japan

**Keywords:** Social behaviour, Human behaviour

## Abstract

Endogenous testosterone promotes behaviours intended to enhance social dominance. However, recent research suggests that testosterone enhances strategic social behaviour rather than dominance seeking behaviour. This possibility has not been tested in a population whose members are known to vary in social status. Here, we explored the relationship between pre-existing social status and salivary testosterone level among members of a rugby team at a Japanese university, where a strong seniority norm maintains hierarchical relationships. Participants played a series of one-shot Ultimatum Games (UG) both as proposer and responder. Opponents were anonymised but of known seniority. We analysed participants’ acquiescence (how much more they offered beyond the lowest offer they would accept). The results showed that, among the most senior participants, higher testosterone was associated with lower acquiescence. Conversely, higher testosterone among the lower-status participants was associated with higher acquiescence. Our results suggest that testosterone may enhance socially dominant behaviour among high-status persons, but strategic submission to seniority among lower-status persons.

## Introduction

Testosterone is a steroid hormone associated with aggression and dominance both in animals and humans. While the mechanisms through which testosterone functions in animals have been well documented^1^, the effects of testosterone on human social behaviour are not fully understood. What is commonly agreed among behavioural endocrinologists is that testosterone is related to sensitivity to status-related cues^2–5^. Eisenegger, Haushofer, and Fehr^6^ contend that testosterone promotes status- and dominance-seeking motives in human social interaction, and thus plays an important role in modulating behaviour in status hierarchies. A meta-analysis of testosterone studies in humans by Archer^2^ also reveals a consistent association between testosterone levels and various measures of dominance, especially when the actor’s status is challenged.

The aim of the present study is to examine how the link between testosterone and status-seeking motives affects the economic decision making of the members of a pre-existing and hierarchically-organised group. This question has implications for neuroendocrinology and social neuroeconomics, since recent studies suggest that testosterone is related to strategic social behaviour, and that the conception of androgens as drivers of socially dominant and aggressive behaviour is too simplistic^7, 8^. This recent theory on the role of testosterone predicts that, among persons ranked lower in a social hierarchy, testosterone will foster non-aggressive, socially obedient behaviour; in contrast, among those ranked more highly, testosterone will induce socially dominant and aggressive behaviour. To test these predictions, we employed the paradigm of economic game experiments, which is known to be useful in disentangling motivations during social interactions^9^. The ultimatum game (UG^10^) is particularly suitable for this purpose. The UG is typically considered a measure of fairness-seeking behaviour, but it is also proposed to measure dominance-seeking behaviour in response to a challenge^11^. The UG pits two players against one another: a “proposer” offers a proportion of a monetary endowment to the “responder”. The responder decides whether to accept or reject the offer. If the responder accepts the offer, the responder receives the money offered by the proposer while the proposer receives the balance. If the responder rejects the offer, neither player receives any money. A rational and self-interested proposer should make a small offer, and a rational and self-interested responder should accept it^12^. Nevertheless, a substantial proportion of proposers make equitable offers and responders frequently reject small offers, at least in Western or market-integrated societies^13, 14^.

Burnham^11^ showed that lower offers in the UG were more often rejected by men with high levels of salivary testosterone, and suggested that the effect could be mediated by testosterone’s impact on status-asserting behaviour: low offers are interpreted by responders as a challenge to their status, and this challenge is more salient to responders high in testosterone. This finding was replicated by Mehta and Beer^15^ both in men and women, but other studies have reported no statistically significant effect of exogenous testosterone on the rejection of inequitable offers^16–19^.

The direction of effects of testosterone on offer behaviour is also inconsistent. Zak *et al*.^18^ report that men who are administered testosterone rather than a placebo offer significantly less money. Conversely, Eisenegger *et al*.^17^ has shown that women administered testosterone offer significantly more than those who receive a placebo. Burnham^11^ showed that male proposers constrained to offer their male opponent either $25 or $5 out of an endowment of $40, and who choose to offer $25, have higher average salivary testosterone levels than those who choose to offer $5, though the difference was not statistically significant. Eisenegger *et al*.^17^ attributed their finding to the status-preserving motives of proposers administered high levels of testosterone. Because a rejected offer may lower the proposer’s status, high levels of testosterone may motivate proposers to avoid circumstances likely to lead to rejection.

The inconsistent effects of testosterone on offer and rejection behaviour may be due to several other factors, including sex (i.e., male sample^11, 15, 16, 18^ vs. female sample^15, 17, 19^), and whether testosterone is endogenous or exogenous (i.e., measured salivary testosterone^11, 15^ vs. administered testosterone^16–19^). Another possibility is that subtle cues of interdependency influence the responder’s rejection behaviour. For example, Declerck, Kiyonari, and Boone^20^ showed that small offers were rejected more frequently when responders were informed that they would be matched with proposers *after* deciding to accept or reject. The authors suggest that their responders may have wished to signal to potential opponents their intention to reject small offers (for an effect of decision timing in a different economic game, see ref. 21).

More generally, sensitivity to environmental cues is known to play a crucial role in human decision making, such that the same economic game may be perceived to represent different challenges depending on the players’ sensitivity to particular environmental cues^22, 23^. We propose that the inconsistent findings with respect to testosterone and aggression may be due to varying cues that enhance or reduce players’ perception of challenges to status. These cues may moderate the relationship between testosterone and proposal/rejection behaviours in a typical laboratory experiment in which there are no a priori status differences between anonymously matched participants who do not know each other. In such experimental situations, the effects of individual differences in sensitivity to status cues unrelated to testosterone levels may obscure the independent effect of testosterone.

Boksem *et al*.^24^ suggest that testosterone affects game behaviour differently depending on context, and argue that the effects of testosterone will depend on whether players face an imminent threat to their status. When threat is imminent, high testosterone may enhance competitive and aggressive behaviour. When threat is not imminent—when an individual’s status is not challenged—competitive behaviour may damage the social status of the individual. In such a situation, testosterone may not stimulate aggressive competitive behaviour but rather prosocial behaviour as a means of enhancing reputation.

We consider it likely that the presence or absence of a threat to status explains at least some inconsistencies between the effects of testosterone on behaviour in economic games. Here, we investigate the effect of testosterone on UG behaviour among individuals who are known to one another and who are embedded in a pre-existing status hierarchy. This approach will allow us to determine the effects of testosterone when a player’s status is challenged (i.e., when a high-status responder is faced with a small offer) and when the same player does not face such a challenge (i.e., when making an offer as a proposer). We hypothesise that responders with higher levels of testosterone will be less tolerant of small offers than will responders with lower levels of testosterone. We also test whether testosterone is related to offer behaviour, but make no specific predictions. Testosterone may promote offers that are small^18^ or large^17^.

We also follow Zak *et al*.’s^18, 25^ strategy of calculating the difference between offers made by players in the role of proposer and the offers those same players tolerate as receiver. If proposers make offers that are above the minimum value at which they themselves would reject (their minimum acceptable offer, or MAO), the difference between the offer and MAO may be termed “generosity”^18, 25^. This term is appropriate when players are (or are assumed to be) equals. However, we are interested in the behaviour of participants who differ in status, where lower ranked players may feel obliged to submit to the wishes of their seniors while higher ranked players may feel entitled to dominate their juniors. We contend that, in such a situation, accepting a lower offer than one would make reflects a willingness to yield to the implicit coercion of the other player. Thus, we refer to the difference between a player’s offer and MAO as “acquiescence” rather than “generosity”.

We hypothesise that acquiescence will parallel the status differences between proposers and responders: lower ranked players will acquiescence more than higher ranked players. The relationship between testosterone and acquiescence is difficult to predict. Zak *et al*.^18^ has shown that higher testosterone is associated with lower acquiescence. However, Beksem *et al*.^24^ argue that testosterone’s effect will depend on the presence or absence of threats, which suggests that this relationship may not hold in the case of the lower ranked players.

## Methods

### Participants

We recruited seventy male undergraduate members of the rugby team of Aoyama Gakuin University (age range, 18–23 years). Twelve participants were first years, 22 were second years, 14 were third years, and 22 were fourth years.

Official sports teams at Japanese universities are organised according to a strict hierarchy based on seniority (cf., refs 26, 27). The fourth year students have the highest seniority and it is customary for junior team members to acquiesce to the demands of senior members. Seniority is based entirely on time spent as a member of the team and not on sporting performance. Given the lack of opportunity for team members to improve their status by displaying superior sporting ability or competing with teammates, the best strategy for the junior members is likely to be to acquiesce to the seniors’ demands until they themselves acquire seniority.

Participants received a show-up reward of JPY 500 and were informed that they would receive extra money depending on their decisions in the experiment.

### Study protocol

All participants took part at the same time. We ran the study in a large conference room before the rugby team’s regular practice. We collected saliva samples from participants both before (~9:10 am) and after (~9:50 am) they played the UGs. Participants delivered 5 ml saliva into a cryotube via passive drool. Saliva specimens were immediately frozen and stored at −80 °C and later assayed in LC-MS/MS.

About four hours after playing the UGs, participants returned to complete a questionnaire and to receive payment. During the interval we calculated participants’ rewards based on their decisions.

We also measured the length of each participant’s second and fourth fingers to calculate their digit ratio (2D:4D), and took facial photographs to permit measurement of facial width to height ratio (fWHR). Findings related to these measures are reported in the Supplementary Information (SI).

The study protocol was approved by the Ethics Committee of Aoyama Gakuin University, and the study was carried out in accordance with the approved protocol which met the requirements of the Declaration of Helsinki. All participants gave written informed consent.

### One-shot Ultimatum Game (UG)

Participants first received instructions about the rules of the UG. They were informed that they would play the UG four times, each time with a different opponent. Each game would consist of two stages, and the participant would play one stage as proposer and one as responder. Participants were further informed that they would receive a supplement to their show-up reward, based on their responses during a randomly selected two of the four games. We ensured that participants fully understood the procedure for calculating the supplementary reward. The supplementary reward was based on the results of two randomly selected games of the four total games, and the value of the reward corresponded to the real values specified in the games. We refer the reader to the SI for further details of the instructions given to participants. While playing the games, participants indicated their choices in a booklet, which they hid from the view of the other participants using a cardboard fence.

Acting as proposer, each participant decided how to divide a monetary endowment between himself and a matched anonymous responder. Proposers decided upon a sum to offer the responder that ranged between nothing and JPY 1000, at increments of JPY 100. As responder, participants indicated whether they would accept or reject the matched proposer’s offer if it was at each of the 11 possible levels (ranging from JPY 1000 for the proposer and nothing for the responder, to nothing for the proposer and JPY 1000 for the responder). The responder was not made aware of the actual offer made by the proposer. This method of measuring the responder’s choices is termed the strategy method. If the responder rejects at the level corresponding to the actual offer made by the proposer, both proposer and responder receive no money. If the responder accepts at the actual offer level, the players receive funds as proposed. For each participant, we calculated minimum acceptable offer (MAO) based on their responses to the 11 possible levels. If a participant showed a non-linear response (for example, rejecting an offer of JPY 100, accepting an offer of JPY 200, but rejecting an offer of JPY 300) we could not calculate his MAO. Non-linear responses were omitted from corresponding analyses and so the numbers of participants vary across game types (See Table 1). A lower MAO indicates tolerance of lower offers.

**Table 1:** Means (standard deviation) of MAO, offer, and acquiescence in the four games.

Year	No information	Peer-to-peer	First year	Fourth year	Total
**MAO**					
First (N = 12)	263.64 (168.95)*	291.67 (156.43)	200.00 (134.84)	191.67 (116.45)	236.81 (122.96)
Second (N = 20)	315.00 (178.52)	270.00 (202.87)	310.00 (207.49)	300.00 (188.56)*	295.00 (172.75)
Third (N = 13)	307.69 (180.10)	292.31 (180.10)	323.08 (183.28)	246.15 (166.41)	292.31 (164.69)
Fourth (N = 22)	300.00 (200.00)*	309.52 (214.25)*	310.00 (249.00)^#^	252.38 (206.44)*	302.27 (193.17)
Total	300.00 (181.14)	290.91 (191.13)	292.31 (206.39)	253.85 (179.48)	286.44 (168.75)
**Offer**					
First (N = 12)	383.33 (169.67)	358.33 (137.90)	441.67 (156.43)	450.00 (178.38)	408.33 (141.96)
Second (N = 22)	440.91 (140.27)	427.27 (148.59)	422.73 (182.40)	450.00 (189.61)	435.23 (145.50)
Third (N = 14)	392.86 (197.93)	378.57 (188.84)	328.57 (197.79)	421.43 (188.84)	380.36 (175.46)
Fourth (N = 22)	322.73 (202.21)	350.00 (187.08)	290.91 (197.39)	290.91 (204.49)	313.64 (162.15)
Total	384.29 (180.69)	381.43 (167.93)	365.71 (193.28)	394.29 (201.36)	381.43 (161.16)
**Acquiescence**					
First (N = 12)	118.18 (204.05)*	66.67 (88.76)	241.67 (150.50)	258.33 (167.65)	172.92 (114.05)
Second (N = 20)	125.00 (174.34)	165.00 (227.75)	105.00 (291.05)	178.95 (263.69)*	141.25 (191.13)
Third (N = 13)	69.23 (118.21)	69.23 (118.21)	−15.38 (114.35)	161.54 (180.46)	71.15 (101.98)
Fourth (N = 22)	38.10 (251.94)*	33.33 (135.40)*	−20.00 (228.50)^#^	52.38 (218.22)*	26.14 (157.83)
Total	84.62 (198.61)	86.36 (166.30)	67.69 (238.56)	149.23 (225.78)	95.52 (161.08)

Some participants responded non-linearly when indicating which offers they would accept. We therefore could not calculate these participants’ MAO and acquiescence, and so they were excluded from the relevant analyses. *Indicates that one participant was excluded, and ^#^indicates that two participants were excluded due to a non-linear response.

**Figure 1:**
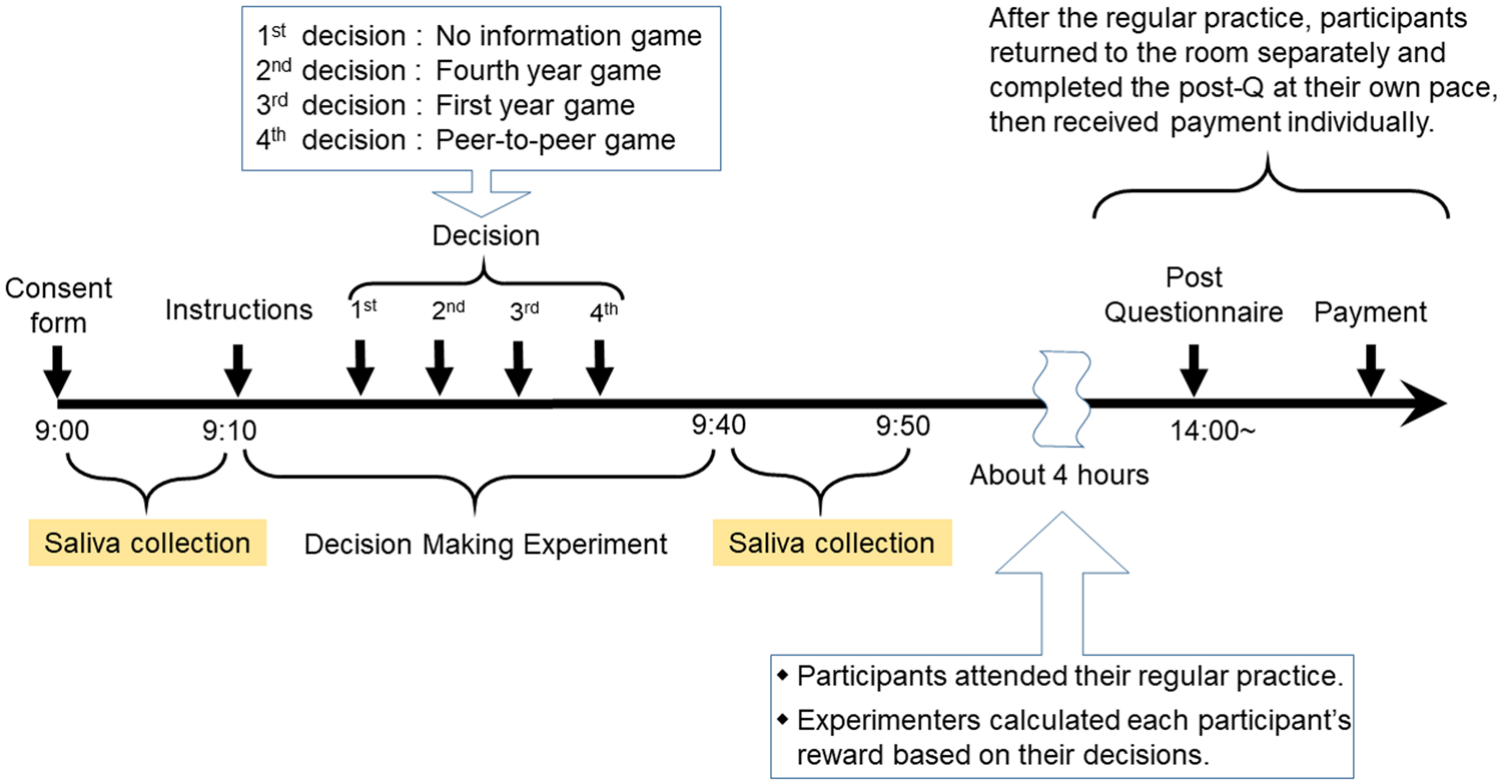
A summary of the experimental protocol.

Two of the four games were played between players with no stated status difference. One of these games was the “*no information game*”: participants played without knowing the seniority of their opponents. The second game was the “*peer-to-peer game*”: participants were informed that they were playing the game with an opponent whose seniority was identical to their own, although the opponent’s identity was concealed. In the other two games, we assessed participants’ behaviour when status differences were known. In one of the two games (“*fourth year game*”), we informed a player in their fourth year that they were playing the game with an opponent in years 1–3. The fourth year player was not informed of the specific seniority level of his opponent, but knew that the opponent was of lower status. The player in years 1–3 was informed that their opponent was a fourth year. In the other game (“*first year game*”), we informed a player in their first year that they were playing the game with an opponent in years 2–4. The first year player was not informed of the specific seniority level of his opponent, but knew that the opponent was senior. The player in years 2–4 was informed that his opponent was a first year. Before each game commenced, an experimenter publicly announced which game was about to be played. Therefore, all participants were aware which groups were playing against one another in each game. Everyone played the same game at the same time (Fig. 1), and therefore the order in which the games were played was the same for all participants: *no information, fourth year, first year, peer-to-peer*.

Because the number of participants differed from year to year (e.g. in the fourth year game, a smaller group of fourth years had to play against opponents drawn from a larger combined group of year 1–3 participants), it was not possible to form one-to-one pairs across years. To ensure that all games were genuinely played between individuals, we matched participants from the smaller group with multiple participants in the larger group. In the case of the fourth year game, each of the first, second, and third years would be paid supplements according to their own choices and their matched fourth year opponent’s choice. The fourth year would be paid a supplement according to his choice and the choice of one of his opponents, selected at random. We informed participants that all pairs chosen for payment would be genuine pairings.

## Results

### Salivary testosterone

We used the pre-game measure of testosterone to analyse the relationship between testosterone and game behaviour, because the post-game measure may be affected by performance during the UG. How participants’ behaviour in the UG affects the post-game measure and the change in testosterone between measures is an interesting question but does not speak to the primary research aims of this study. Therefore, we report findings concerning the post-game measure and the change score in the SI. We used testosterone value after log-transformation (pmol/L). In our analyses we coded seniority as a categorical variable with categories 1, 2, 3 and 4. We found no statistically significant differences in the baseline levels of testosterone (pre-T) as a function of participant seniority, *F*(3, 66) = 0.99, *p* = 0.40, η_p_^2^ = 0.04 (see also additional information in the Table S7).

### Minimum Acceptable Offers (MAO)

We first tested if game type and participant seniority affected tolerance of small offers by conducting a general linear model analysis with MAO as the dependent variable, game type as a repeated factor, and seniority as a between-participants factor. We found a significant main effect of game type, *F*(3, 177) = 3.29, *p* = 0.022, ω_g_^2^ = 0.008. No other effect was significant (Table S1). The mean levels of MAO in the four games, which can be seen in Table 1, indicate that participants are generally more tolerant of small offers in the fourth year game, regardless of whether they are a junior participant playing against a fourth year or a fourth year playing against a junior opponent. We then analysed how testosterone levels were related to MAO with a general linear model analysis in which testosterone, seniority, game type, and their interactions were entered as independent variables, but found no statistically significant main or interaction effects of testosterone (Table S2).

### Offers

**Figure 2:**
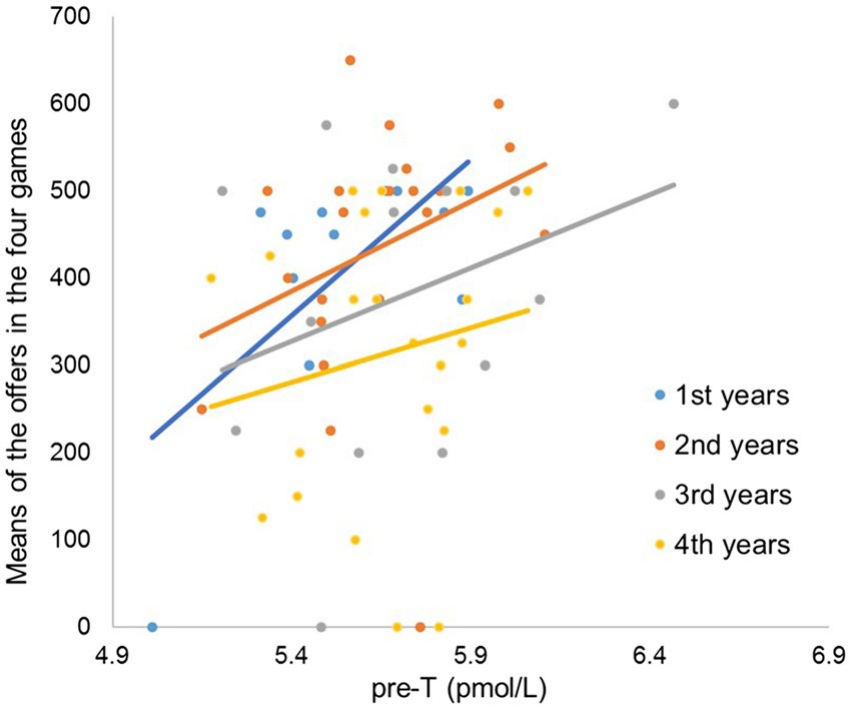
The relationship between baseline testosterone level and the mean of the offers in the four games for each seniority level. Spearman’s correlation coefficient: first years (*r*_*s*_ = 0.50, *p* = 0.09), second years (*r*_*s*_ = 0.45, *p* = 0.04), third years (*r*_*s*_ = 0.28, *p* = 0.32), fourth years (*r*_*s*_ = 0.18, *p* = 0.41).

We analysed the effects of game type and seniority on offer level using a general linear model analysis (Table S3). No effect was significant, although the main effect of seniority was marginally significant, *F*(3, 66) = 2.36, *p* = 0.080, ω_g_^2^ = 0.041, as was the interaction between game type and seniority, *F*(9, 198) = 1.67, *p* = 0.099, ω_g_^2^ = 0.007. Senior players, and especially fourth years, tended to offer less (Table 1). We next performed a general linear model analysis of offers by seniority, game type, and testosterone (Table S4). The main effect of testosterone was highly significant, *F*(1, 62) = 8.43, *p* = 0.005. None of the other main effects or interactions were statistically significant. Figure 2 shows the relationship between testosterone and the mean offers made across the four games, split by seniority. Regardless of seniority, participants with higher rather than lower levels of testosterone offered more money, *r*_*s*_ = 0.28, *p* = 0.02.

### Acquiescence

**Figure 3:**
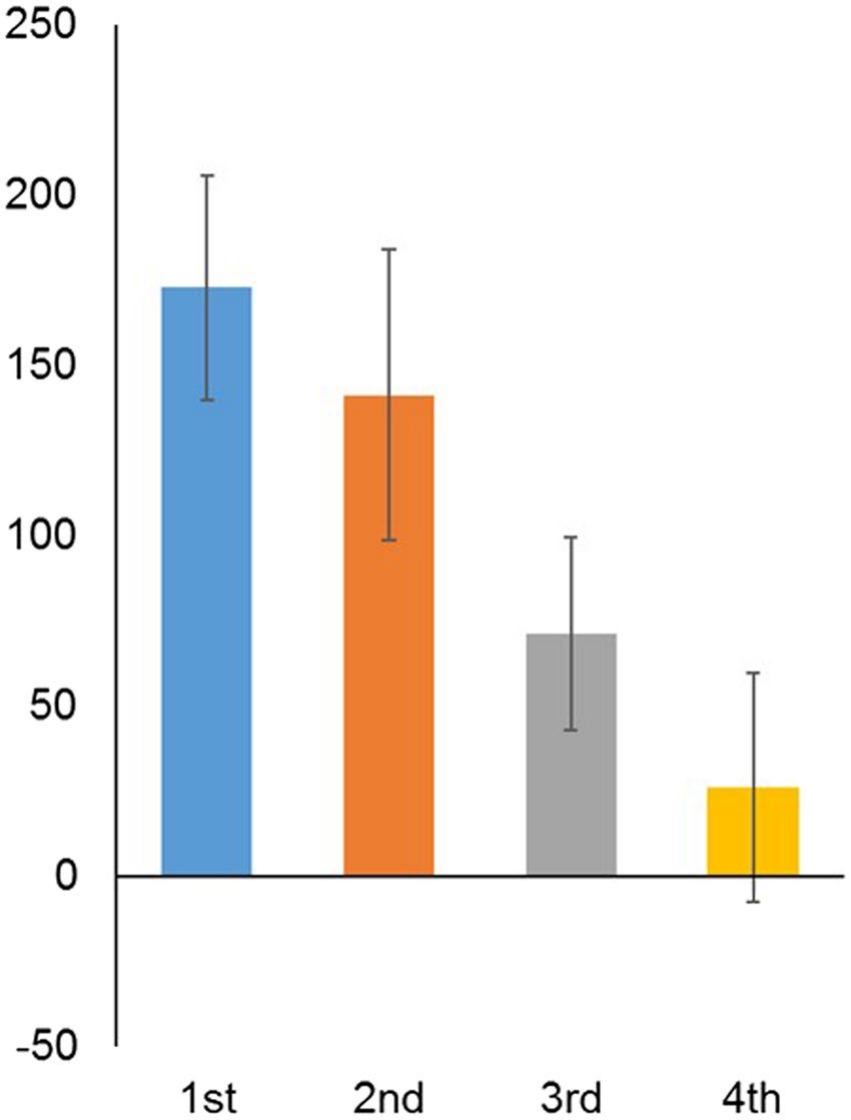
Mean acquiescence (Y axis) in each year (X axis). Error bars represent standard errors of the mean.

On average, participants’ offers (JPY 381.43) exceeded their MAOs (JPY 286.44), *t*(66) = 4.34, *p* < 0.0001, *d* = 0.55. The main effect of seniority on acquiescence (offer minus MAO) was significant, *F*(3, 59) = 2.94, *p* = 0.040, ω_g_^2^ = 0.052 (Fig. 3, Table S5). Across all game types, first years acquiesced most, followed by second and third years. Participants in their first, *t*(11) = 5.25, *p* < 0.001, second, *t*(19) = 3.30, *p* = 0.004, and third years, *t*(12) = 2.52, *p* = 0.027, acquiesced significantly more than zero. Fourth years’ acquiescence was not significantly different from zero, *t*(21) = 0.78, *p* = 0.446.

Game type also had a significant effect on acquiescence, *F*(3, 177) = 4.00, *p* = 0.009, ω_g_^2^ = 0.019. The overall levels of acquiescence in the fourth year game were greater than in the other three game types (Table 1). The interaction between seniority and game type was marginally significant, *F*(9, 177) = 1.67, *p* = 0.098, ω_g_^2^ = 0.013. A post hoc Tukey test revealed that, in the first year game, the first and third years differed significantly (*p* = 0.041), as did the first and fourth years (*p* = 0.018). The first years acquiesced more than the senior players. In the fourth year game, a post hoc Tukey test showed that the fourth years and the first years differed marginally (*p* = 0.089). In the peer-to-peer game, the second years and the fourth years differed significantly (*p* = 0.049).

**Figure 4:**
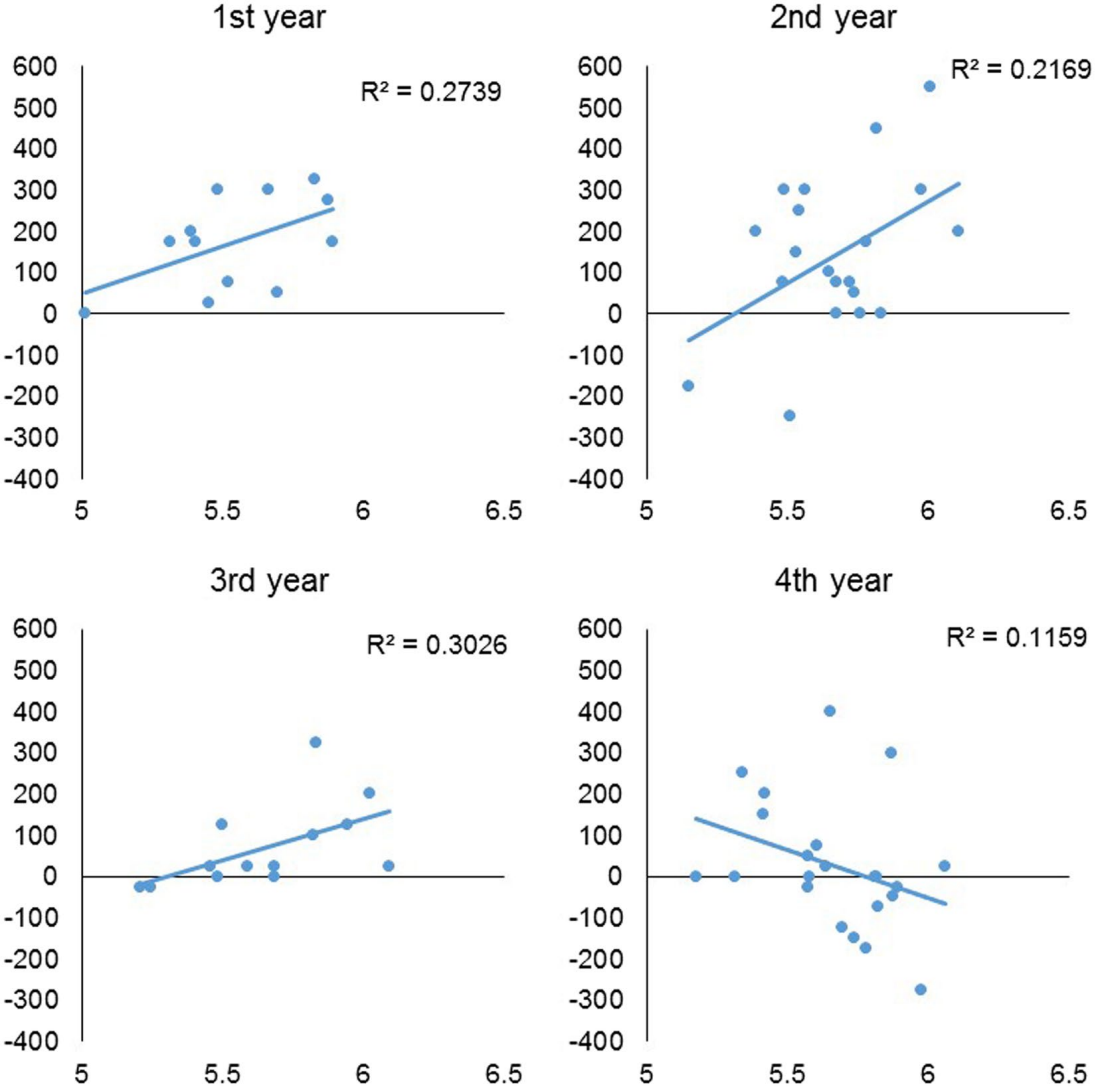
Plot of the Pre-T (pmol/L) levels (X-axis) and overall levels of acquiescence (Y-axis) in each year. R-squares were calculated from univariate linear regression analysis. Spearman’s correlation coefficient: first years (*r*_*s*_ = 0.40, *p* = 0.20), second years (*r*_*s*_ = 0.28, *p* = 0.24), third years (*r*_*s*_ = 0.68, *p* = 0.01), fourth years (*r*_*s*_ = −0.40, *p* = 0.07).

A general linear model analysis of acquiescence by testosterone, game type, seniority, and their interactions revealed a marginally significant main effect of testosterone, *F*(1, 55) = 3.56, *p* = 0.065. The main effect of seniority was significant, *F*(3, 55) = 3.70, *p* = 0.017, and testosterone and seniority interacted significantly, *F*(3, 55) = 3.97, *p* = 0.012. There were no significant main or interaction effects of game type. Figure 4 shows how the relationship between testosterone and overall acquiescence (the mean of acquiescence across the four games) varied according to the seniority of the participant. Among the first, second, and third years, testosterone was related to acquiescence in a similar way: higher testosterone was associated with more generous offers. However, this relationship was reversed among the fourth years: higher testosterone was associated with lower acquiescence (see also Table S6).

## Discussion

This study is the first to demonstrate that endogenous testosterone is related to strategic social decision making that differs according to the decision maker’s status, by utilising an appropriate dependent variable measured with an economic game (i.e., “acquiescence” in the UG) with participants who belong to a strong pre-established social hierarchy. In this study we examined how men who are chronically embedded in hierarchical relationships as members of a university rugby team make decisions in a series of ultimatum games (UGs), assessing behaviour using three measures: minimum acceptable offer (MAO), offer, and acquiescence (offer minus MAO).

Overall, junior participants exhibited a lower MAO—a tolerance of lower offers—when they knew their opponent was senior. However, when the level of testosterone was considered in the analysis, we found no significant effects of game type, seniority, or testosterone on MAO. The prediction that high testosterone would promote the rejection of unfair offers, based on the findings of earlier studies^11, 15^, was not confirmed. However, our results are in line with those of other studies that reported no effect of testosterone on rejection in the UG^16, 17, 19^. We found a strong main effect of testosterone on offer level, which is consistent with Eisenegger *et al*.^17^, but contrary to Zak *et al*.^18^. We found no effect of seniority on offer when we included level of testosterone in analyses.

However, when we considered the difference between offer and MAO, which we term “acquiescence”, we saw that the effect of testosterone differed as a function of seniority, consistent with recent theory^7, 8^ on the roles of testosterone in strategic social interactions: that testosterone induces dominant behaviour among higher ranking individuals and obedience or submissiveness among lower ranking individuals. We found no main or interaction effects of game type on acquiescence, but did find a significant interaction between seniority and testosterone. Senior players acquiesced *less* if their testosterone was high rather than low, while junior players acquiesced *more* if their testosterone was high rather than low. This interaction effect between testosterone and social status among persons embedded in hierarchical relationships is a novel finding, which suggests the interesting possibility that testosterone is implicated in behaviour that could be characterised as tactical rather than dominant.

Only fourth year students with high testosterone showed a typically dominant response. It is possible that the different effects of testosterone on the behaviour of junior and senior players reflect the players’ unequal status in day-to-day interactions, where the junior players have no opportunity to dominate those who are senior. This interpretation is in line with the prediction that testosterone promotes “strategic” rather than simply aggressive/dominant behaviour. Although we requested that participants keep their decisions secret and we assured them that responses would remain anonymous, they may have expected that any discrepancies in the rewards received by participants at different seniority levels would reveal behaviour inconsistent with that expected by the hierarchy (alternatively, players may simply have thought that behaving in a manner contrary to that demanded by their status would be inappropriate or immoral). If so, the junior players may not have interpreted a low offer as a challenge to their status and therefore a high level of testosterone among these players may have promoted prosocial behaviour or strategic submission for the sake of maintaining the proper functioning of the hierarchy, either to enhance team performance or to preserve their own ability to benefit from a senior status in coming years. The most senior players, however, behave according to their expected roles, with predictable effects of testosterone on acquiescence.

It remains unclear why we did not find any interaction effects of seniority and testosterone on MAO and offer when conducting separate analyses. One possibility is that these measures are less revealing of a person’s overall behaviour than a combined measure that reveals both the degree of generosity towards others and the tolerance for unfair treatment at the hands of others. We should not expect offer size or tolerance of inequitable offers to fully capture the concept of acquiescence when considered independently. Our participants played both the roles of proposer and responder in each game. Our within-participants design allowed us to identify each player’s level of acquiescence independent of their beliefs about other players’ responses to inequitable offers. For example, a player who rejects any offer lower than 50% and expects that others will respond in the same manner will tend to offer at least 50% of the endowment to their opponent. Another individual who tolerates offers above 30% and expects that others would do the same will tend to make offers above 30%. Although these players differ in their offers and MAOs, in neither case can we say that they are acquiescing in the sense of accepting an offer that they expect others would not accept. Therefore, generating a measure of acquiescence from offer and MAO may permit more useful tests of the effects of testosterone on UG behaviour.

The strategy method, which we employed here, requires participants to play both UG roles without direct knowledge of their opponents’ behaviour. Fehr, Fischbacher, von Rosenbladt, Schupp, and Wagner^28^ speculate that the strategy method may be less emotionally arousing than the more direct method of play, because responders make hypothetical decisions rather than responding directly to a genuine offer. It is plausible, therefore, that testosterone effects may be weaker in response to the strategy game and that we might see stronger effects if play were more direct—an interesting question for future research. However, it would be difficult to test the case of very small offers from the proposer (e.g., JPY 200 for the responder and JPY 800 for the proposer) without using deception, at least in market integrated societies^14^.

When men play economic games before an audience of same-sex peers, they cooperate less, while women cooperate more^29^. In our study, participants played in the presence of other men but their peers were not aware of their responses. Charness and Rustichini^29^ suggest that men may wish to signal their formidability to their peers while women may wish to signal cooperativeness. When men are chronically embedded in hierarchical groups, they may be motivated to signal formidability if they are high ranking and cooperativeness if they are low ranking. Future research could test whether the effects of testosterone on UG behaviour are strengthened when participants play before an audience. In addition to the effect of context, Carré and colleagues demonstrated that exogenous testosterone does not have a simple effect on aggressive behaviour in men, but they highlighted the critical role that individual difference factors play in catalysing the effect of testosterone on human aggression^30^. They used the Point Subtraction Aggression Paradigm^31^ to assay aggressiveness, and found that men who were administered testosterone behaved aggressively, but the effect was restricted to men who scored high in trait dominance or low in trait self-control. The question of how testosterone could moderate the effects of individual differences is also an important issue. Our results may imply that men with high testosterone are more sensitive to status inequality than are men with low testosterone, irrespective of their own dominance status. Further research will be needed to determine which individual difference factors moderate the effect of testosterone.

Zak *et al*.^18^ reported lower acquiescence (which they termed “generosity”) when their participants were administered testosterone rather than a placebo. Our results partially replicate Zak *et al*.^18^, as we report similar behaviour among senior players but not among junior players, suggesting that exogenous testosterone may have different effects on players who are unknown to one another, compared to players who are embedded in a pre-existing status hierarchy. The causal relationship between testosterone and behaviour can also only be elucidated by experimental studies in which testosterone is directly manipulated^16^. Although studying exogenous testosterone effects may elucidate the causal relationships, there is also an opportunity for further development of the methodology. For example, Zak *et al*. tested their participants 16 hours after administering testosterone, based on evidence from studies of hypogonadal males^32^. On the other hand, a recent study^33^ assessing healthy young men showed that blood testosterone levels peak at three hours after administration of testosterone gel. There remains the possibility that the results of Zak *et al*.’s study may have been different if tests had been performed at the presumed true peak testosterone level. Nevertheless, one interesting topic for future study would be to compare the effects of exogenous testosterone on generosity/acquiescence among players embedded in strict hierarchical relationships, such as members of Japanese university sports teams or of military forces, with the effects among players of similar rank but who are nevertheless freer to compete for status.

### Electronic supplementary material

Supplementary Information
