# Author Correction: Testosterone promotes either dominance or submissiveness in the Ultimatum Game depending on players’ social rank

**DOI:** 10.1038/s41598-023-46112-0

**Published:** 2023-11-21

**Authors:** Yukako Inoue, Taiki Takahashi, Robert P. Burriss, Sakura Arai, Toshikazu Hasegawa, Toshio Yamagishi, Toko Kiyonari

**Affiliations:** 1 Department of Cognitive and Behavioral Science, Graduate School of Arts and Sciences, The University of Tokyo, Tokyo, 153-8902 Japan; 2 Graduate School of Letters, Hokkaido University, Sapporo, 060-0810 Japan; 3 Faculty of Psychology, Basel University, 4055 Basel, Switzerland; 4 Department of Psychological and Brain Sciences, University of California, Santa Barbara, CA 93106 USA; 5 Brain Science Institute, Tamagawa University, Machida, 194-8610 Japan; 6 Graduate School of International Corporate Strategy, Hitotsubashi University, Tokyo, 101-8439 Japan; 7 School of Social Informatics, Aoyama Gakuin University, Sagamihara, 252-5258 Japan

Correction to: *Scientific Reports* 10.1038/s41598-017-05603-7, published online 13 July 2017

The original version of this Article contained errors. An incorrect molecular weight was used to convert testosterone concentrations from pg/ML to pmol/L. Since the error was only in the conversion ratio, all originally reported patterns remain unchanged. There are no changes in the F-value, p-value of the general linear model, and rank correlation coefficient (except one rounding error in the legend of Figure 2).

The corrected Figure 2 and Figure 4 and accompanying legends appear below.

**Figure 2:**
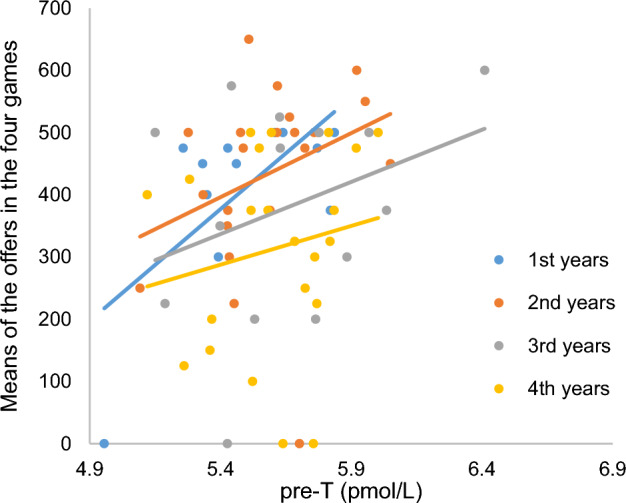
The relationship between baseline testosterone level and the mean of the offers in the four games for each seniority level. Spearman’s correlation coefficient: first years (*r*_*s*_ = 0.50, *p* = 0.09), second years (*r*_*s*_ = 0.45, *p* = 0.04), third years (*r*_*s*_ = 0.2**9**, *p* = 0.32), fourth years (*r*_*s*_ = 0.18, *p* = 0.41).

**Figure 4:**
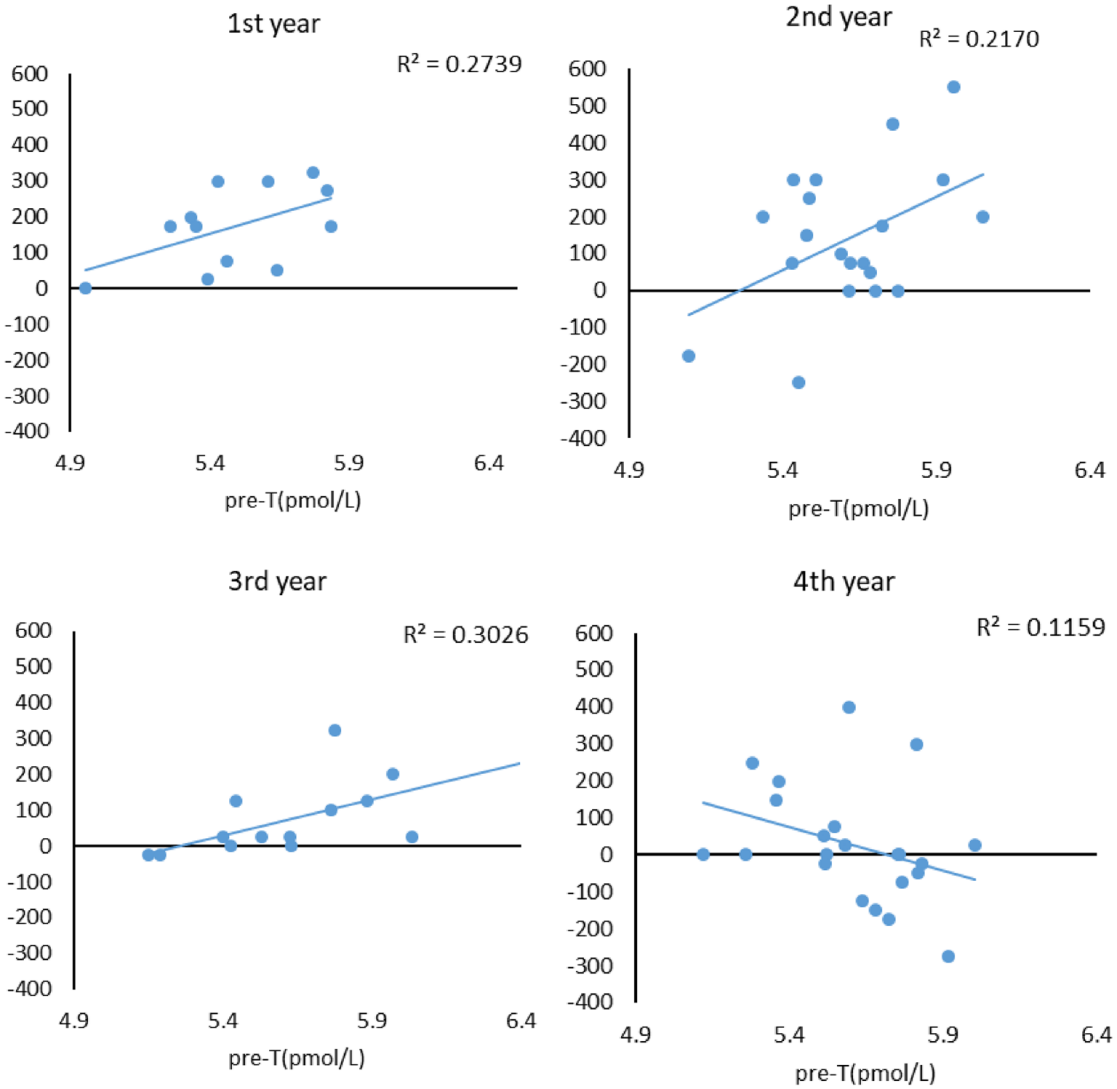
Plot of the Pre-T (pmol/L) levels (X-axis) and overall levels of acquiescence (Y-axis) in each year. R-squares were calculated from univariate linear regression analysis. Spearman’s correlation coefficient: first years (*rs* = 0.40, *p* = 0.20), second years (*rs* = 0.28, *p* = 0.24), third years (*rs* = 0.68, *p* = 0.01), fourth years (*rs* =  − 0.40, *p* = 0.07).

Figure 2 legend,

“The relationship between baseline testosterone level and the mean of the offers in the four games for each seniority level. Spearman’s correlation coefficient: first years (*r*_*s*_ = 0.50, *p* = 0.09), second years (*r*_*s*_ = 0.45, *p* = 0.04), third years (*r*_*s*_ = 0.28, *p* = 0.32), fourth years (*r*_*s*_ = 0.18, *p* = 0.41).”

should read:

“The relationship between baseline testosterone level and the mean of the offers in the four games for each seniority level. Spearman’s correlation coefficient: first years (*r*_*s*_ = 0.50, *p* = 0.09), second years (*r*_*s*_ = 0.45, *p* = 0.04), third years (*r*_*s*_ = 0.2**9**, *p* = 0.32), fourth years (*r*_*s*_ = 0.18, *p* = 0.41).”

Additionally, correcting the conversion changes testosterone values, their log transformation, the sum-of-squares, and the mean squares in the general linear model with testosterone. Note that the differences in Supplementary Figure S5 and S6 are slight.

The corrected Supplementary Figure S5 and S6 appear below.

Figure S5


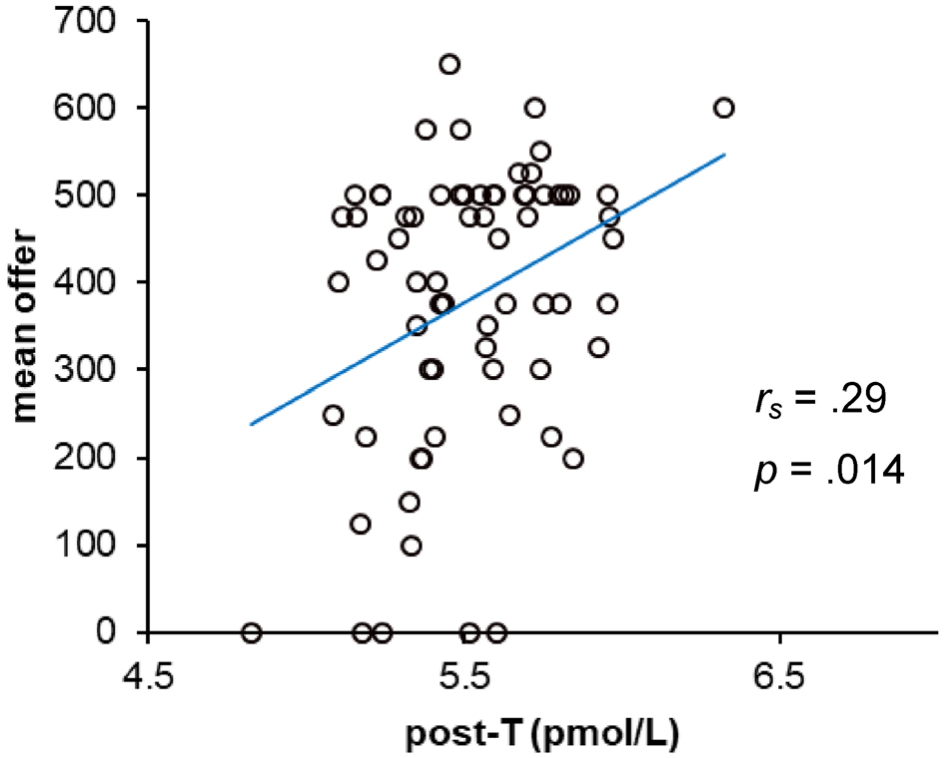


Figure S6
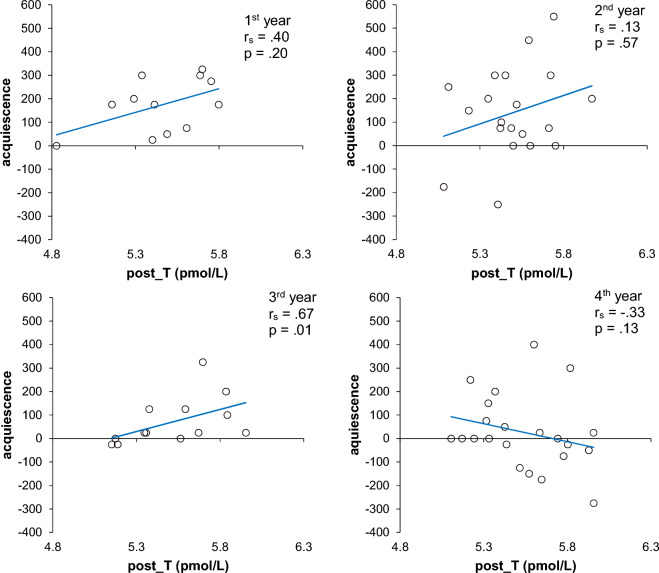


Finally, there were errors in Supplementary Tables S2, S4, S6, S7. In Supplementary Tables S2, S4, S6, Seniority SS, Seniority MS, Game SS, Game MS, Seniority * Game SS, Seniority * Game MS values were incorrect. In Supplementary Table S7, the testosterone values were incorrect. The corrected Supplementary Tables S2, S4, S6, S7 appear below.

Table S2

**Table:** 

Source	df	SS	MS	F	p
Seniority	3	639,576.385	213,192.128	1.92	0.137
pre-T	1	62,131.967	62,131.967	0.56	0.458
Seniority * pre-T	3	628,999.591	209,666.530	1.89	0.142
Error	55	6,102,387.788	110,952.505		
Game	3	587.026	195.675	0.02	0.997
Seniority * Game	9	33,145.988	3682.888	0.33	0.963
Game * pre-T	3	453.225	151.075	0.01	0.998
Seniority * Game * pre-T	9	32,949.182	3661.020	0.33	0.964
Error (Game)	165	1,823,933.284	11,054.141		

Table S4

**Table:** 

Source	df	SS	MS	F	p
Seniority	3	89,532.120	29,844.040	0.33	0.806
pre-T	1	770,189.839	770,189.839	8.43	**0.005**
Seniority * pre-T	3	106,424.439	35,474.813	0.39	0.762
Error	62	5,663,540.833	91,347.433		
Game	3	21,847.764	7282.588	0.63	0.597
Seniority * Game	9	60,507.272	6723.030	0.58	0.812
Game * pre-T	3	23,143.409	7714.470	0.67	0.574
Seniority * Game * pre-T	9	62,496.446	6944.050	0.60	0.796
Error (Game)	186	2,152,620.564	11,573.229		

Table S6

**Table:** 

Source	df	SS	MS	F	p
Seniority	3	924,870.579	308,290.193	3.70	**0.017**
pre-T	1	296,373.599	296,373.599	3.56	0.065
Seniority * pre-T	3	993,142.525	331,047.508	3.97	**0.012**
Error	55	4,582,326.073	83,315.020		
Game	3	29,304.166	9768.055	0.42	0.739
Seniority * Game	9	146,551.653	16,283.517	0.70	0.708
Game * pre-T	3	33,362.036	11,120.679	0.48	0.698
Seniority * Game * pre-T	9	153,985.712	17,109.524	0.74	0.675
Error (Game)	165	3,834,362.776	23,238.562		

Table S7

**Table:** 

Year	n	Mean (SD)					
		Pre-T (n = 70)	Post-T (n = 70)	Change-T (n = 70)	R2D:4D (n = 69)	L2D:4D (n = 69)	fWHR (n = 67)
1st	12	5.49 (0.26)	5.46 (0.28)	− 0.03 (0.09)	0.9331 (0.0285)	0.9308 (0.0304)	2.2445 (0.1136)
2nd	22	5.59 (0.23)	5.49 (0.22)	-0.10 (0.11)	0.9360 (0.0333)	0.9342 (0.0312)	2.1963 (0.1904)
3rd	14	5.66 (0.34)	5.58 (0.34)	-0.08 (0.10)	0.9376 (0.0258)	0.9298 (0.0127)	2.2181 (0.1783)
4th	22	5.61 (0.23)	5.54 (0.27)	-0.07 (0.13)	0.9342 (0.0340)	0.9254 (0.0335)	2.2774 (0.1889)
F		0.99	0.55	0.92	0.06	0.33	0.79
*η*_*p*_^*2*^		0.04	0.02	0.04	0.00	0.02	0.04
*p* Value		0.40	0.65	0.44	0.98	0.80	0.50

These errors do not affect the conclusions of the Article.

