# Supplementary material for: Testosterone promotes either dominance or submissiveness in the Ultimatum Game depending on players’ social rank
Source: Sci Rep. 2017 Jul 13;7:5335. doi: 10.1038/s41598-017-05603-7 (PMC5509644; doi:10.1038/s41598-017-05603-7)
Supplement: Supplementary file 1 — Supplementary Information [file 41598_2017_5603_MOESM1_ESM.pdf]

Supplementary Information

Testosterone promotes either dominance or submissiveness in the Ultimatum Game depending on players' social rank

Yukako Inoue, Taiki Takahashi, Robert P. Burriss, Sakura Arai, Toshikazu Hasegawa, Toshio Yamagishi, & Toko Kiyonari

Details of the general linear model analysis in the main text

First, we provide tables to report more detailed information about the general linear model analysis reported in the main text. Although the data analyses we report in both the main text and SI were generated using SAS 9.4., we calculated the effect size of generalized omega squared using the free statistical software HAD (Shimizu, 2016).

Table S1. Details of the general linear model analysis with Mean Acceptable Offer (MAO) as the dependent variable, game type as a repeated factor, and seniority as a between-participants factor.

Source	*df*	*SS*	*MS*	*F*	ω_g^2	*p*
Seniority	3	166523.228	55507.743	0.48	.000	.696
Error (Seniority)	59	6787365.661	115040.096			
Game	3	103671.477	34557.159	3.29	.008	**.022**
Seniority * Game	9	89198.553	9910.950	0.94	.000	.488
Error (Game)	177	1857547.479	10494.619			

Table S2. Details of the general linear model analysis with MAO as the dependent variable, and testosterone, seniority, game type, and their interactions entered as independent variables.

Source	*df*	*SS*	*MS*	*F*	*p*
Seniority	3	639478.639	213159.546	1.92	.137
pre-T	1	62131.967	62131.967	0.56	.458
Seniority * pre-T	3	628999.591	209666.53	1.89	.142
Error	55	6102387.788	110952.505		
Game	3	583.735	194.578	0.02	.997
Seniority * Game	9	33143.101	3682.567	0.33	.963
Game * pre-T	3	453.225	151.075	0.01	.998
Seniority * Game * pre-T	9	32949.182	3661.02	0.33	.964
Error (Game)	165	1823933.284	11054.141		

Table S3. Details of the general linear model analysis with offer as the dependent variable, game type as a repeated factor, and seniority as a between-participants factor.

Source	*df*	*SS*	*MS*	*F*	ω_g^2	*p*
Seniority	3	693937.229	231312.41	2.36	.041	**.080**
Error (Seniority)	66	6474491.342	98098.354			
Game	3	36739.039	12246.346	1.08	.000	.357
Seniority * Game	9	169608.225	18845.358	1.67	.007	**.099**
Error (Game)	198	2235963.203	11292.743			

Table S4. Details of the general linear model analysis with offer as the dependent variable; testosterone, seniority, game type, and their interactions were entered as independent variables.

Source	*df*	*SS*	*MS*	*F*	*p*
Seniority	3	89689.702	29896.567	0.33	.806
pre-T	1	770189.839	770189.839	8.43	**.005**
Seniority * pre-T	3	106424.439	35474.813	0.39	.762
Error	62	5663540.833	91347.433		
Game	3	21860.315	7286.772	0.63	.597
Seniority * Game	9	60525.046	6725.005	0.58	.812
Game * pre-T	3	23143.409	7714.47	0.67	.574
Seniority * Game * pre-T	9	62496.446	6944.05	0.60	.796
Error (game)	186	2152620.564	11573.229		

Table S5. Details of the general linear model analysis with acquiescence as the dependent variable, game type as a repeated factor, and seniority as a between-participants factor.

Source	*df*	*SS*	*MS*	*F*	ω_g^2	*p*
Seniority	3	875506.329	291835.443	2.94	.052	**.040**
Error (Seniority)	59	5848065.099	99119.747			
Game	3	272884.203	90961.401	4.00	.019	**.009**
Seniority * Game	9	342702.728	38078.081	1.67	.013	**.098**
Error (Game)	177	4025789.336	22744.573			

Table S6. Details of the general linear model analysis with acquiescence as the dependent variable; testosterone, seniority, game type, and their interactions were entered as independent variables.

Source	*df*	*SS*	*MS*	*F*	*p*
Seniority	3	925544.763	308514.921	3.70	**.017**
pre-T	1	296373.599	296373.599	3.56	**.065**
Seniority * pre-T	3	993142.525	331047.508	3.97	**.012**
Error	55	4582326.073	83315.02		
Game	3	29339.712	9779.904	0.42	.738
Seniority * Game	9	146622.002	16291.334	0.70	.707
Game * pre-T	3	33362.036	11120.679	0.48	.698
Seniority * Game * pre-T	9	153985.712	17109.524	0.74	.675
Error (game)	165	3834362.776	23238.562		

Other variables investigated in this study

From here we report the results of our tests involving other independent variables and testosterone indices: post-measured testosterone (post-T), change levels of testosterone (change-T = post-T minus pre-T), right hand digit ratio (R2D:4D), left hand digit ratio (L2D:4D) and facial width-to-height-ratio (fWHR). 2D:4D is a marker for prenatal testosterone levels (Manning, 2002) and is implicated in social behaviour (van Honk et al., 2011). fWHR may be sexually dimorphic (Weston et al. 2007, but see Lefevre et al. 2012) with men possessing relatively shorter lower faces relative to their width than do women, and it has been linked with dominance and aggression in men (Carré & McCormick, 2008; Stirrat & Perrett, 2010).

Most of the participants consented to a body measurement session that took place approximately two months prior to the experimental session. Measurements included a facial photograph and hand scans, as well as measures of grip power and body weight, which we do not analyze here. Photographs were taken with a SONY alpha55 SLT-A55VY digital camera on a tripod while participants sat upright in front of a blue background under standardised lighting. Using ImageJ (NIH open-source software) we measured fWHR (n=67) by dividing the bizygomatic width (maximum horizontal distance from the left facial boundary to the right facial boundary at the cheekbones) by the upper-face height (vertical distance from the mid-point of the upper-lip to the highest point of the epicanthic fold or eyelid crease as applicable). Left and right hand 2D:4D (n=69) were measured from image scans of each hand by taking the length of the index and ring fingers from the ventral proximal crease to the tip of the finger.

Table S7 shows the average of six testosterone indices and the standard deviation for participants at each seniority level. There were no significant differences between seniority levels within each index. Change levels of testosterone are depicted in the Figure S1 in each grade. As shown in Figure S1, change levels decreased overall from before to after the experiment. Except for the first years, the average levels of change-T significantly differed from zero.

Table S7. Salivary testosterone levels after log-transformation (pmol/L) in pre-T, post-T, and change-T and both hands' 2D:4D and fWHR for players at each seniority level. A one-way between-participants ANOVA was conducted to compare the effect of seniority on each testosterone index shown in the lower row.

		Mean (SD)					
Year	n	pre-T (n=70)	post-T (n=70)	change-T (n=70)	R2D:4D (n=69)	L2D:4D (n=69)	fWHR (n=67)
1st	12	5.54 (0.26)	5.51 (0.28)	- 0.03 (0.09)	.9331 (.0285)	.9308 (.0304)	2.2445 (.1136)
2nd	22	5.65 (0.23)	5.55 (0.22)	- 0.10 (0.11)	.9360 (.0333)	.9342 (.0312)	2.1963 (.1904)
3rd	14	5.72 (0.34)	5.63 (0.34)	- 0.08 (0.10)	.9376 (.0258)	.9298 (.0127)	2.2181 (.1783)
4th	22	5.66 (0.23)	5.60 (0.27)	- 0.07 (0.13)	.9342 (.0340)	.9254 (.0335)	2.2774 (.1889)
F		.99	.55	.92	.06	.33	.79
η_p^2		.04	.02	.04	.00	.02	.04
p value		.40	.65	.44	.98	.80	.50

(note) We failed to collect one participant's hand scans (2nd year student) and three of the participants' facial photos (two 2nd year students and one 3rd year student).

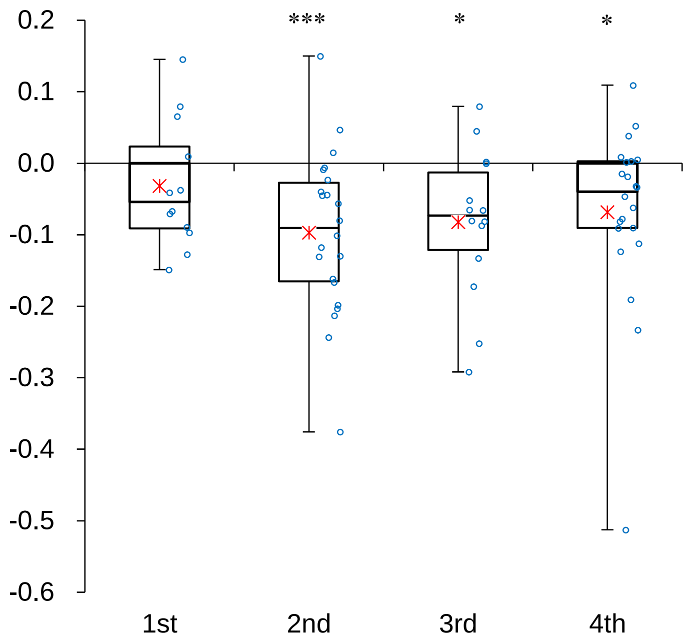

Figure S1. The Y axis shows the change levels of testosterone from before to after the experiment for players at each seniority level (X axis). Positive values indicate increase and negative values indicate decrement. Asterisks indicate the statistical significance of t tests for difference from zero (* $p < .05$, *** $p < .001$).

Table S8 shows values of the Spearman correlation coefficient for the relationship between each testosterone index. Across salivary testosterone indices, pre- and post- measured testosterone showed strong positive and significant correlations. Conversely, change levels showed smaller or moderate correlations with pre- and post-measures. These three salivary testosterone indices did not show statistically significant correlations with the other indices. Right hand 2D:4D correlated strongly with left hand 2D:4D and both also showed moderate correlations with fWHR in the predicted direction.

Table S8. Values of the Spearman correlation coefficients for each testosterone index. Sample sizes vary due to missing values: saliva samples (n = 70), R2D:4D (n = 69), L2D:4D (n = 69), fWHR (n = 67).

	pre-T	post-T	change-T	R2D:4D	L2D:4D
post-T	**.89*** **				
change-T	– .18	**.23†**			
R2D:4D	– .08	– .06	.10		
L2D:4D	– .18	– .19	– .00	**.60*** **	
fWHR	.02	.08	.11	**– .27***	**– .25***

(† p<.10, * p<.05, ** p<.01, *** p<.0001)

Association between other indices of testosterone and rejection in the UG

In the main text, we report the association between pre-T and MAO. Here we report the association between post measured testosterone/changes in levels of testosterone and mean MAOs across the four game types. Participants who did not show a linear rejection threshold were excluded from the relevant analyses. We investigated how rejection thresholds across the four games affected testosterone levels by conducting two-way ANOVAs to test for differences in post-T and change-T based on means of MAO, seniority level, and their interaction (Table S9). We found no statistically significant main or interaction effects of post-T. Similarly, we tested if MAO and seniority affected change in level of testosterone, and found a significant main effect of MAO, $F(1, 59) = 4.75$, $p = .033$. No other effect was significant. As shown in Figure S2, change in level of testosterone was positively correlated with mean MAO. This suggests that participants who tended to reject smaller offers across all four partner conditions (i.e., participants who tended to indicate large minimum acceptable offers) maintain or increase their testosterone levels by the end of the experiment.

Table S9. Post-T or change-T were the dependent variables and participant seniority and their mean MAO over four games were the independent variables.

DV: Post-T

Source	*df*	*SS*	*MS*	*F*	η_p^2	*p*
Seniority	3	0.14718437	0.04906146	0.76	.037	.523
mean MAO	1	0.1054028	0.1054028	1.63	.027	.207
Seniority * mean MAO	3	0.20344147	0.06781382	1.05	.051	.379
Error	59	3.82522147	0.06483426			
Corrected total	66	4.21039262				

DV: Change-T

Source	*df*	*SS*	*MS*	*F*	η_p^2	*p*
Seniority	3	0.01734286	0.00578095	0.50	.025	.685
mean MAO	1	0.05510046	0.05510046	4.75	.074	**.033**
Seniority * mean MAO	3	0.00636589	0.00212196	0.18	.009	.908
Error	59	0.68510348	0.01161192			
Corrected total	66	0.81735644				

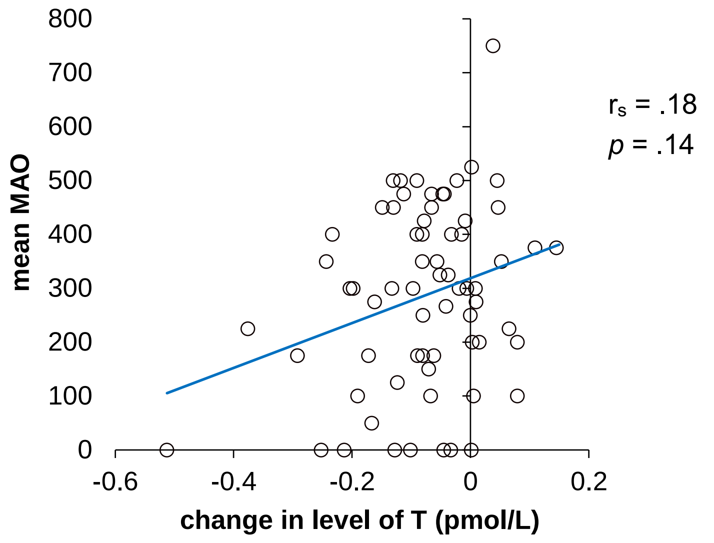

Figure S2. Scatter plot of change in testosterone and mean MAO

We also examined the effect of other previously measured indices of testosterone in a general linear model analysis of MAO by seniority, game type, testosterone index (right hand 2D:4D, left hand 2D:4D, and fWHR), and their interactions. Since there were no effects of game type, we examined mean MAO, as reported in the Table S10. Right hand 2D:4D interacted significantly with seniority on mean MAO. fWHR had a marginally significant main effect on mean MAO. Figure S3 illustrates the association between right 2D:4D and mean MAO over each seniority level. Figure S4 shows the association between fWHR and mean MAO.

Table S10. Tests of the effects of various testosterone indices (i.e., R2D:4D, L2D:4D, fWHR) on mean MAO.

Source	*df*	*SS*	*MS*	*F*	η_p^2	*p*
Seniority	3	255013.179	85004.393	3.31	.146	**.026**
R2D4D	1	16079.791	16079.791	0.63	.011	.432
Seniority * R2D4D	3	257740.289	85913.430	3.35	.148	**.025**
Error	58	1487840.229	25652.418			
Corrected total	65	1796128.998				

Source	*df*	*SS*	*MS*	*F*	η_p^2	*p*
Seniority	3	62717.625	20905.875	0.73	.036	.540
L2D4D	1	362.090	362.090	0.01	.000	.911
Seniority * L2D4D	3	65586.104	21862.035	0.76	.038	.520
Error	58	1665783.931	28720.413			
Corrected total	65	1796128.998				

Source	*df*	*SS*	*MS*	*F*	η_p^2	*p*
Seniority	3	6876.598	2292.199	0.08	.004	.971
fWHR	1	103981.569	103981.569	3.60	.061	**.063**
Seniority * fWHR	3	6099.530	2033.177	0.07	.004	.976
Error	56	1615304.078	28844.716			
Corrected total	63	1792464.193				

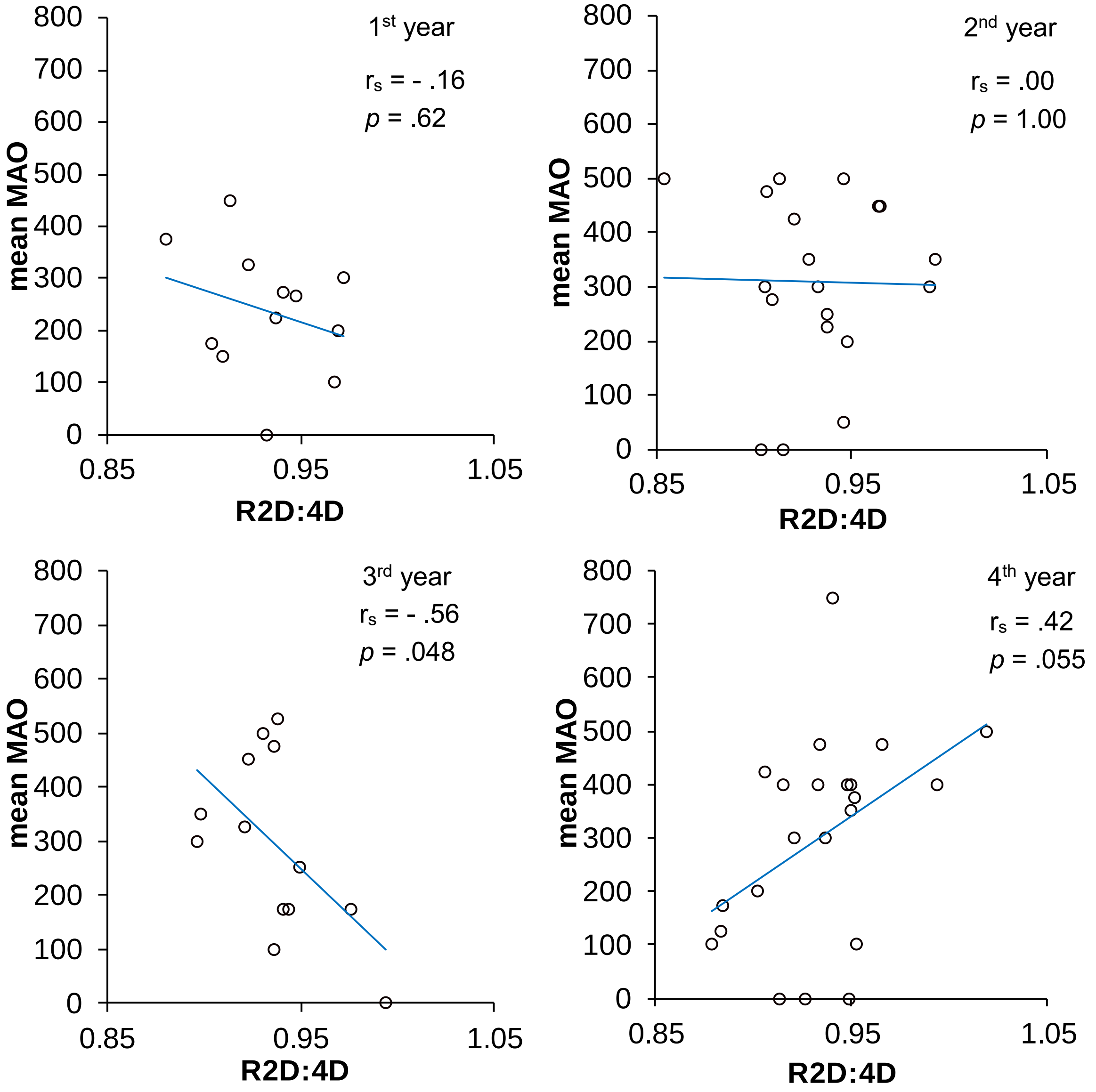

Figure S3. Scatter plot of right hand 2D:4D against mean MAO at each seniority level.

As shown in Figure S3, mean MAO and right 2D:4D were positively correlated among fourth years, which means that senior participants who are likely to have been exposed to high levels of testosterone prenatally tended to accept smaller offers on average than participants whose exposure was low. However, junior participants showed null or negative correlations between mean MAO and right 2D:4D. Figure S4 illustrates the positive correlation between mean MAO and fWHR, indicating that those with a physical cue to high testosterone tended to reject smaller offers than those without a cue to high testosterone.

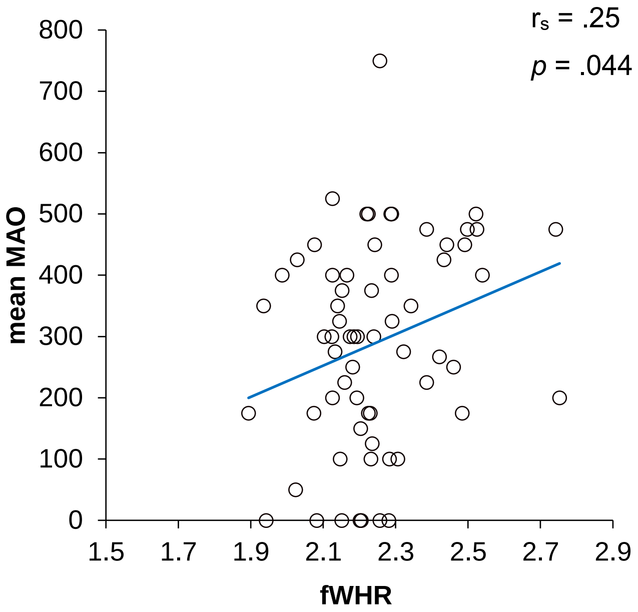

Figure S4. Scatter plot of fWHR and mean MAO

To summarize, pre-T and post-T have no effect on rejection thresholds, but change-T is positively correlated with MAO, indicating that those who rejected smaller offers tended to maintain or increase their salivary testosterone levels between playing the UGs. R2D:4D shows inconsistent patterns across seniority levels. fWHR shows patterns consistent with expectations, though the effects were marginal when we considered the effect of seniority in a general linear model analysis.

Association with testosterone and amount of offer in the UG

We conducted two-way ANOVAs to test for post-T and change-T effects on mean offer and seniority (Table S11). Mean offer had a significant main effect on levels of post-T. As shown in Figure S5, those who offered more across the four games showed higher levels of post-T than those who offered less. The pattern was similar to that for pre-T, as reported in the main text. The interaction between seniority and mean offer on change-T was marginally significant. However, post hoc Tukey-Kramer least square tests showed no significant effect in either combination.

Table S11. Post-T or change-T were the dependent variables and participant seniority and mean offer over four games were the independent variables.

DV: Post-T

Source	*df*	*SS*	*MS*	*F*	η_p^2	*p*
Seniority	3	0.20304805	0.06768268	1.08	.050	.366
mean offer	1	0.86250415	0.86250415	13.72	.181	**.001**
Seniority * mean offer	3	0.17703505	0.05901168	0.94	.044	.427
Error	62	3.89686662	0.06285269			
Corrected total	69	4.94609911				

DV: Change-T

Source	*df*	*SS*	*MS*	*F*	η_p^2	*p*
Seniority	3	0.05048613	0.01682871	1.45	.065	.238
mean offer	1	0.02118036	0.02118036	1.82	.029	.182
Seniority * mean offer	3	0.08513336	0.02837779	2.44	.106	**.073**
Error	62	0.72117202	0.01163181			
Corrected total	69	0.86965666				

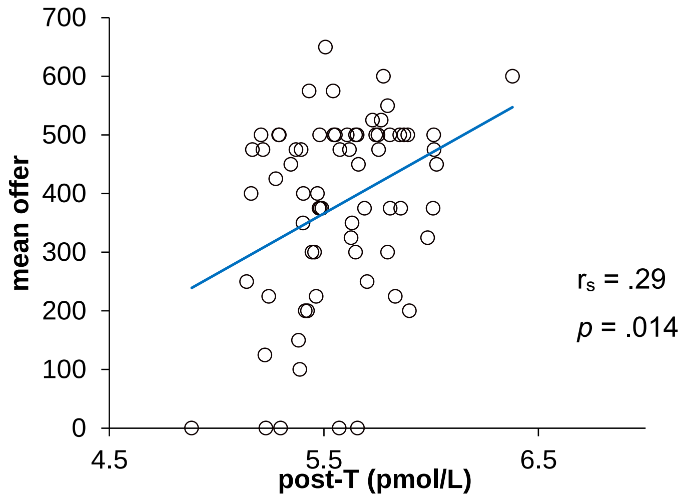

Figure S5. Scatter plot illustrating the relationship between mean offer and post-T.

We also separately examined the effect of other indices of testosterone in a general linear model analysis of offers by seniority, game type, testosterone index (i.e., right hand 2D:4D or left hand 2D:4D or fWHR), and their interactions. No significant main effect or interaction effect was found for right 2D:4D. There was also no significant main effect of left 2D:4D, but a three-way interaction effect between game type, seniority, and left 2D:4D was significant, though difficult to explain. No significant main effect or interaction effect was found for fWHR. When we combined four games into one (i.e., mean offer), none of the significant main effects or interaction effects remained (Table S12).

Table S12. Tests of the effects of various testosterone indices (i.e., R2D:4D, L2D:4D, fWHR) on mean offer.

Source	*df*	*SS*	*MS*	*F*	η_p^2	*p*
Seniority	3	130856.184	43618.728	1.82	.082	.154
R2D4D	1	7338.978	7338.978	0.31	.005	.582
Seniority * R2D4D	3	130290.847	43430.282	1.81	.082	.155
Error	61	1465034.036	24016.951			
Corrected total	68	1785380.435				

Source	*df*	*SS*	*MS*	*F*	η_p^2	*p*
Seniority	3	53670.280	17890.093	0.71	.034	.549
L2D4D	1	190.651	190.651	0.01	.000	.931
Seniority * L2D4D	3	60139.693	20046.564	0.80	.038	.501
Error	61	1535530.768	25172.636			
Corrected total	68	1785380.435				

Source	*df*	*SS*	*MS*	*F*	η_p^2	*p*
Seniority	3	53850.427	17950.142	0.69	.034	.562
fWHR	1	29717.288	29717.288	1.14	.019	.290
Seniority * fWHR	3	42319.902	14106.634	0.54	.027	.656
Error	59	1537081.498	26052.229			
Corrected total	66	1776679.104				

Association between acquiescence and post-T or change-T

To test whether mean acquiescence affected the levels of post-T or change-T, we conducted two-way ANOVAs.

Table S13. Post-T and change-T were the dependent variables and participants' seniority and their mean acquiescence across the four game types was the independent variable.

DV: Post-T

Source	*df*	*SS*	*MS*	*F*	η_p^2	*p*
Seniority	3	0.33437795	0.11145932	1.86	.086	.147
Acquiescence	1	0.32895495	0.32895495	5.48	.085	**.023**
Seniority * Acquiescence	3	0.53089911	0.17696637	2.95	.130	**.040**
Error	59	3.54430288	0.06007293			
Corrected total	66	4.21039262				

DV: Change-T

Source	*df*	*SS*	*MS*	*F*	η_p^2	*p*
Seniority	3	0.00478232	0.00159411	0.13	.007	.942
Acquiescence	1	0.00486401	0.00486401	0.40	.007	.531
Seniority * Acquiescence	3	0.02875902	0.00958634	0.78	.038	.509
Error	59	0.72314603	0.01225671			
Corrected total	66	0.81735644				

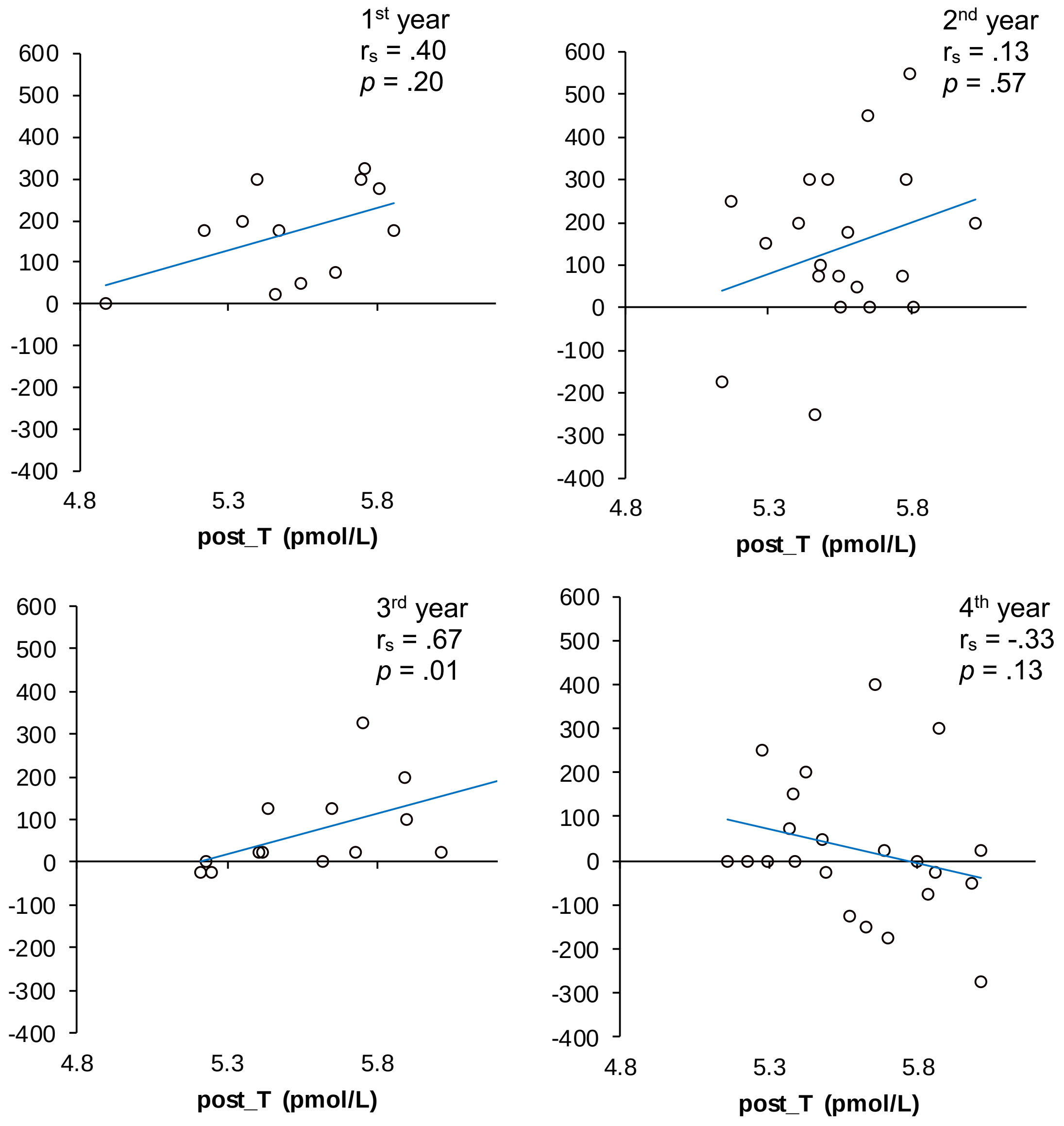

Figure S6. Scatter plot of the Post-T levels (X-axis) and mean acquiescence (Y-axis) for participants in each seniority level.

As shown in Table S13, there was a significant interaction effect of seniority and mean acquiescence on levels of post-T but not change-T. Scatter plots of post-T and mean acquiescence at each seniority level (Figure S6) indicate that post-T and mean acquiescence positively correlate in junior players. Only the fourth years showed the opposite pattern. These patterns were the same as that observed between pre-T and acquiescence, as reported in the main text. The change levels of testosterone were not

predicted significantly by mean acquiescence or the interaction with seniority.

As shown in Table S14, none of the remaining testosterone indices had any significant effect on mean acquiescence, nor did they interact with seniority.

Table S14. Mean acquiescence was the dependent variable and participant seniority and each testosterone index (i.e., R2D:4D, L2D:4D, fWHR) were independent variables.

Source	*df*	*SS*	*MS*	*F*	η_p^2	*p*
Seniority	3	20956.571	6985.524	0.29	.015	.836
R2D4D	1	14471.860	14471.860	0.59	.010	.445
Seniority * R2D4D	3	25119.665	8373.222	0.34	.017	.795
Error	58	1418575.254	24458.194			
Corrected total	65	1669962.121				

Source	*df*	*SS*	*MS*	*F*	η_p^2	*p*
Seniority	3	101979.870	33993.290	1.47	.071	.232
L2D4D	1	19056.185	19056.185	0.83	.014	.367
Seniority * L2D4D	3	110732.734	36910.911	1.60	.076	.200
Error	58	1339413.531	23093.337			
Corrected total	65	1669962.121				

Source	*df*	*SS*	*MS*	*F*	η_p^2	*p*
Seniority	3	108377.175	36125.725	1.50	.075	.224
fWHR	1	7203.831	7203.831	0.30	.005	.586
Seniority * fWHR	3	94153.382	31384.461	1.31	.065	.282
Error	56	1346618.329	24046.756			
Corrected total	63	1658593.750				

Reference

Carré, J. M., & McCormick, C. M. (2008). In your face: facial metrics predict aggressive behaviour in the laboratory and in varsity and professional hockey players. *Proceedings of the Royal Society of London B: Biological Sciences, 275*(1651), 2651-2656. doi:10.1098/rspb.2008.0873

Lefevre, C. E., Lewis, G. J., Bates, T. C., Dzhelyova, M., Coetzee, V., Deary, I. J., & Perrett, D. I. (2012). No evidence for sexual dimorphism of facial width-to-height ratio in four large adult samples. *Evolution and Human Behavior, 33*(6), 623-627. doi:10.1016/j.evolhumbehav.2012.03.002

Manning, J. T. (2002). Digit ratio: A pointer to fertility, behavior, and health. Rutgers University Press.

Shimizu, H. (2016). An introduction to the statistical free software HAD: Suggestions to improve teaching, learning and practice data analysis. *Journal of Media, Information and Communication*, *1*, 59-73. (in Japanese) http://norimune.net/696

Stirrat, M., & Perrett, D. I. (2010). Valid facial cues to cooperation and trust male facial width and trustworthiness. *Psychological Science, 21*(3), 349-354. doi 0.1177/0956797610362647

Van Honk, J., Schutter, D. J., Bos, P. A., Kruijt, A. W., Lentjes, E. G., & Baron-Cohen, S. (2011). Testosterone administration impairs cognitive empathy in women depending on second-to-fourth digit ratio. *Proceedings of the National Academy of Sciences, 108*(8), 3448-3452. doi:10.1073/pnas.1011891108

Weston, E. M., Friday, A. E., & Lio, P. (2007). Biometric evidence that sexual selection has shaped the hominin face. *PLoS ONE, 2*(8): e710. doi:10.1371/journal.pone.0000710

[Experimental materials]

There follow translations of participant instruction sheets (#1), and decision sheets for each condition and tailored for each level of participant seniority (#2 ~ #5).

#1: Instructions for all participants.

#2: Decision sheet in the first condition (No information game) for all participants.

#3-: Decision sheet in the second condition (Fourth year game).

  #3-1: For junior students (i.e., 1st to 3rd year students).

  #3-2: For the 4th year students.

#4-: Decision sheet in the third condition (First year game).

  #4-1: For the 1st year students.

  #4-2: For the senior students (i.e., 2nd to 4th year students).

#5: Decision sheet in the fourth condition (Peer-to-peer game) for all participants.

#1
Instructions for all participants

ID Number

Instructions For The Experiment On Social Interaction

Introduction

- During the experiment please follow the experimenters' directions.
- Talking with others is strictly prohibited.
- Please enter your ID number in the box provided.
- Once everyone is ready the experimenter will explain the experiment. Please follow along carefully as the experimenter reads through the explanation starting on the next page.
- If during the explanation there is anything you do not understand or if you have other questions, please do not hesitate to raise your hand and let the experimenter know.

General Outline of the Experiment

- This is an experiment involving a type of monetary transaction.
- The amount of your earnings in the experiment will ultimately vary according to decisions made by each person in the monetary transaction.
- The money you earn in this experiment **will be paid to you today in cash at the end of your rugby practice**.
- For each monetary transaction you will be paired with one of the other players.
- One person in each pair will be ***proposer*** and the other will be ***recipient*** of the proposal. (A more detailed explanation will follow.)
- In all, the transaction will be repeated four times.
- You will be paired with a different person for each transaction.

- The total reward money you earn today will be calculated from the results of 2 of the 4 transactions.
- Which transactions are used for the calculation will be determined by lottery at the end of the experiment.
- However, it is possible that, depending on the results of the transactions, the reward will be ¥0. Therefore, we promise that we will pay everyone today's minimum guaranteed amount of ¥500 as show up fee, without exception.
- Whether you can earn extra money above and beyond the ¥500 will depend on the results of your monetary transactions.
- None of you will find out whom you are paired with, or who made what decisions during or after the experiment.
- Everyone's anonymity will be completely maintained, so please relax and freely make whatever decisions you like.

Next, let us explain about the “monetary transaction” in this experiment in more detail.

[About The “Monetary Transaction”]

1. The “monetary transaction” takes place between two people, a *proposer* and a *recipient*.

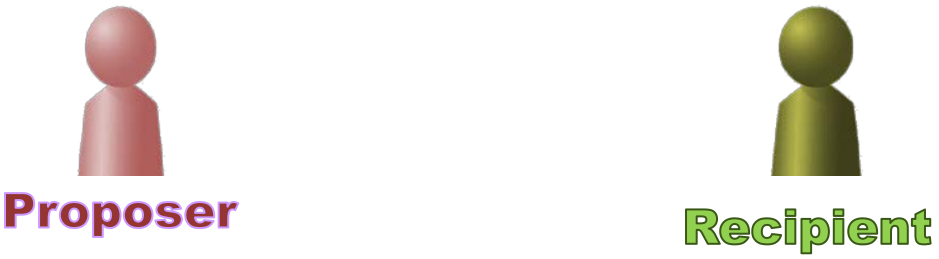

2. The experimenters give the proposer ¥1000 to fund the monetary transaction.

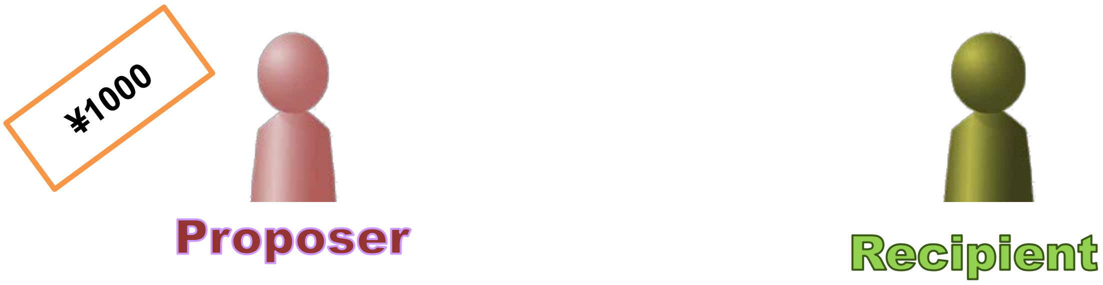

3. The proposer considers how to split the ¥1000 with the recipient and makes a proposal.

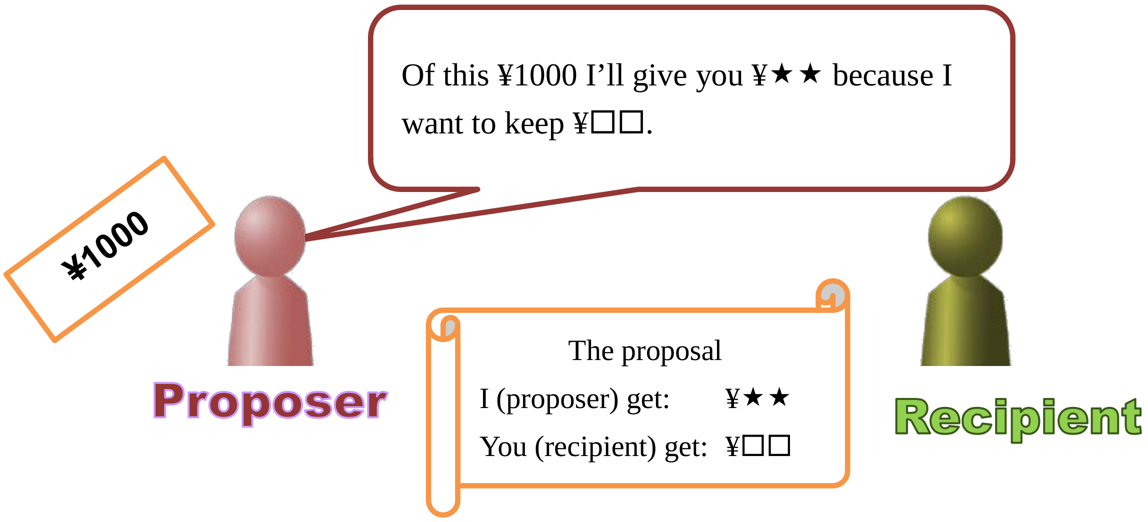

4. The recipient decides to either **accept or reject** the proposed split.

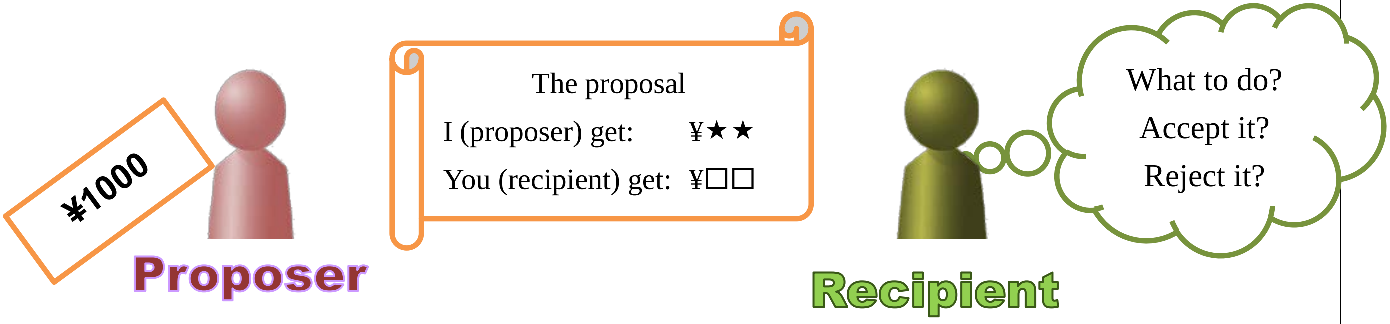

5. If the recipient accepts the proposal ...

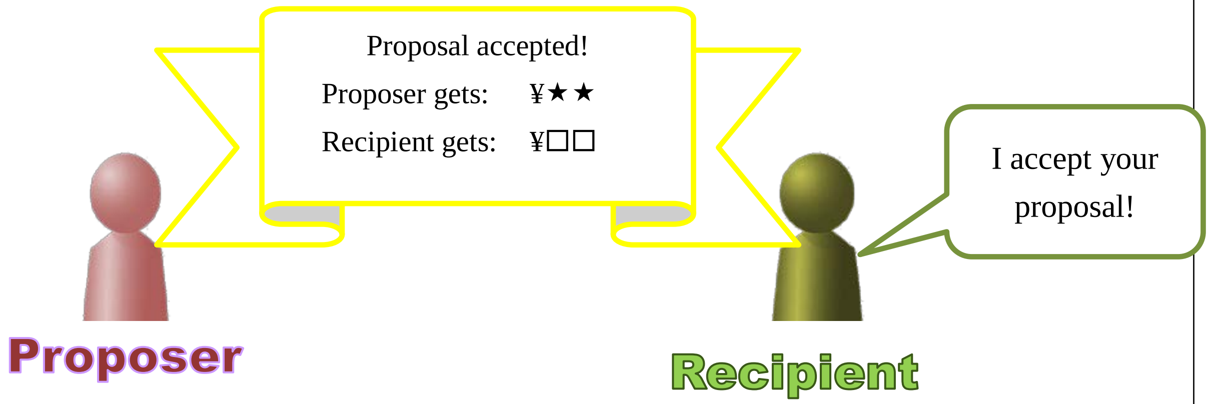

➔ Each receives the proposed amount.

6. If the recipient does not like the proposed amount and rejects it...

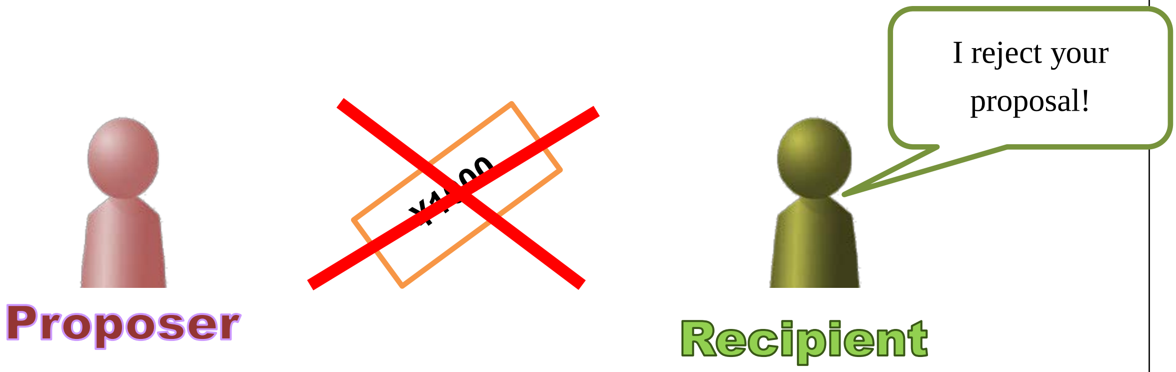

➔ **Both lose all the money and get no reward money for this transaction. (¥0 for each.)**

Next, here are more details about your transaction partners.

[About your transaction partners]

1. We will run the monetary transactions four times.
2. **For every transaction you will be paired with a different person and never with the same person twice.**
3. Which player will be the proposer and which player the recipient in each transaction has not been decided yet.
4. At the end of the experiment lotteries will be held to determine which role each person will play and which 2 transactions will be used to calculate the rewards.
5. Therefore, in every monetary transaction...
   1) **First,** everyone acts **as a proposer** and proposes how to split the ¥1000 with the other player.
   2) The proposal is made in increments of ¥100 from ¥0 to ¥1000.
   3) **Next,** everyone acts **as a recipient** and decides whether to accept or reject the other player's (the proposer's) proposal for that transaction.
   4) However, at that point, the actual split proposed by the other player will not be known. Therefore, you will look at a list of possible ¥1000 splits and decide whether you will accept or reject each of them.
6. This procedure will be performed 4 times, with different pairs of players.

That concludes our explanation of the "monetary transactions" in this experiment. If there is anything that you do not understand, please raise your hand and address your question to the experimenter.

Enter your ID number here.

#2
No information game decision sheet for all participants.

An Experiment on Monetary Transaction
Decision Sheets: First transaction

We now begin the experiment.

- You will be given instructions by the experimenter.
- Complete the task as instructed.
- Read this cover page. Do not move on to the next page until you are instructed to do so.

- Your paired partners in these transactions **will be different in each transaction**.
- **You will not know who your transaction partners are** either during or after the experiment.
- Your transaction partner will be determined by lottery at the end of the experiment.
- Further, which 2 of the 4 transactions will be used for actual reward calculation will be determined by lottery. Your earnings for the day will be the total of a minimum guaranteed ¥500 plus a reward calculated from the results of the 2 transactions selected by lottery.
- We ask that you write your decisions about each transaction on the decision sheets. After all participants have made their decisions, these will be input into a computer to tabulate the results.
- Please wait until the experimenter says to proceed to the next step.
- After completing each transaction, do not turn to the next page. Wait quietly until instructed to continue on to the next page by the experimenter.
- **It is strictly forbidden to try to look at the work of those around you!**

If you have understood the explanation thus far, please wait until instructed to proceed by the experimenter.

You will be paired with one of the other participants.

Please decide what you would do
if you were the ***proposer***.

For this monetary transaction you have been given ¥1000. How would you like to divide it with the other player? Please write your proposal in the following box.

Of ¥1000:

Yourself ¥ ______

Other player ¥ ______

Total ¥ 1000

Please wait until everyone has finished.
However, please take care others cannot see your proposal.

You will be paired with one of the other participants.

Please decide what you would do
if you were the ***recipient***.

Possible proposals the other player may make are listed below. Please decide whether you would accept or reject each and circle the appropriate response.

	If the other player proposed the following, what would you do?				**Your decision** (Circle one)	
1	To other player	¥1000,	**To you**	**¥0**	Accept	Reject
2	To other player	¥900,	**To you**	**¥100**	Accept	Reject
3	To other player	¥800,	**To you**	**¥200**	Accept	Reject
4	To other player	¥700,	**To you**	**¥300**	Accept	Reject
5	To other player	¥600,	**To you**	**¥400**	Accept	Reject
6	To other player	¥500,	**To you**	**¥500**	Accept	Reject
7	To other player	¥400,	**To you**	**¥600**	Accept	Reject
8	To other player	¥300,	**To you**	**¥700**	Accept	Reject
9	To other player	¥200,	**To you**	**¥800**	Accept	Reject
10	To other player	¥100,	**To you**	**¥900**	Accept	Reject
11	To other player	¥0,	**To you**	**¥1000**	Accept	Reject

Please ensure you have not missed any responses and close your booklet.

Take care that others cannot see your work.

Enter your ID number here.

#3-1
Fourth year game decision sheet for 1st to 3rd year students

For 1st – to 3rd –year students

Decision sheets: Second transaction

The second transaction will now begin.

- In this transaction the other player **will be someone different than in the previous transaction**.
- Do not turn the page until instructed to do so by the experimenter.
- During the experiment **it is strictly forbidden to try to look at the work of those around you**.

Please wait until everyone is ready to begin.

Do not turn the page until everyone is instructed to do so by the experimenter.

You will be paired with
one of the 4th –year students.

Please decide what you would do
if you were the ***proposer***.

For this monetary transaction you have been given ¥1000. How would you like to divide it with the other player? Please write your proposal in the following box.

Of ¥1000:	
Yourself	¥ ______
Other player	¥ ______
Total	¥ 1000

Please wait until everyone has finished.
However, please take care others cannot see your proposal.

You will be paired with one of the 4th –year students.

Please decide what you would do if you were the ***recipient***.

Possible proposals the other player may make are listed below. Please decide whether you would accept or reject each and circle the appropriate response.

	If the other player proposed the following, what would you do?	**Your decision** (Circle one)	
1	To other player ¥1000, **To you ¥0**	Accept	Reject
2	To other player ¥900, **To you ¥100**	Accept	Reject
3	To other player ¥800, **To you ¥200**	Accept	Reject
4	To other player ¥700, **To you ¥300**	Accept	Reject
5	To other player ¥600, **To you ¥400**	Accept	Reject
6	To other player ¥500, **To you ¥500**	Accept	Reject
7	To other player ¥400, **To you ¥600**	Accept	Reject
8	To other player ¥300, **To you ¥700**	Accept	Reject
9	To other player ¥200, **To you ¥800**	Accept	Reject
10	To other player ¥100, **To you ¥900**	Accept	Reject
11	To other player ¥0, **To you ¥1000**	Accept	Reject

Please ensure you have not missed any responses and close your booklet.

Take care that others cannot see your work.

Enter your ID number here.

⬇

#3-2
Fourth year game decision sheet for 4th year students.

For 4th –year students

Decision sheets: Second transaction

The second transaction will now begin.

- In this transaction the other player **will be someone different than in the previous transaction**.
- Do not turn the page until instructed to do so by the experimenter.
- During the experiment **it is strictly forbidden to try to look at the work of those around you**.

Please wait until everyone is ready to begin.

Do not turn the page until everyone is instructed to do so by the experimenter.

You will be paired with
one of the 1st to 3rd –year students.

Please decide what you would do
if you were the ***proposer***.

For this monetary transaction you have been given ¥1000. How would you like to divide it with the other player? Please write your proposal in the following box.

Of ¥1000:	
Yourself	¥ ______
Other player	¥ ______
Total	¥ 1000

Please wait until everyone has finished.
However, please take care others cannot see your proposal.

You will be paired with one of the 1st to 3rd –year students.

Please decide what you would do if you were the ***recipient***.

Possible proposals the other player may make are listed below. Please decide whether you would accept or reject each and circle the appropriate response.

	If the other player proposed the following, what would you do?	**Your decision** (Circle one)	
1	To other player ¥1000, **To you ¥0**	Accept	Reject
2	To other player ¥900, **To you ¥100**	Accept	Reject
3	To other player ¥800, **To you ¥200**	Accept	Reject
4	To other player ¥700, **To you ¥300**	Accept	Reject
5	To other player ¥600, **To you ¥400**	Accept	Reject
6	To other player ¥500, **To you ¥500**	Accept	Reject
7	To other player ¥400, **To you ¥600**	Accept	Reject
8	To other player ¥300, **To you ¥700**	Accept	Reject
9	To other player ¥200, **To you ¥800**	Accept	Reject
10	To other player ¥100, **To you ¥900**	Accept	Reject
11	To other player ¥0, **To you ¥1000**	Accept	Reject

Please ensure you have not missed any responses and close your booklet.

Take care that others cannot see your work.

Enter your ID number here.

⬇

#4-1
First year game decision sheet for 1st year students

For 1st –year students

Decision sheets: Third transaction

The third transaction will now begin.

- In this transaction the other player **will be someone different than in the previous transaction**.
- Do not turn the page until instructed to do so by the experimenter.
- During the experiment **it is strictly forbidden to try to look at the work of those around you**.

Please wait until everyone is ready to begin.

Do not turn the page until everyone is instructed to do so by the experimenter.

You will be paired with
one of the 2nd to 4th –year students.

Please decide what you would do
if you were the ***proposer***.

For this monetary transaction you have been given ¥1000. How would you like to divide it with the other player? Please write your proposal in the following box.

Of ¥1000:	
Yourself	¥ ______
Other player	¥ ______
Total	¥ 1000

Please wait until everyone has finished.
However, please take care others cannot see your proposal.

You will be paired with one of the 2nd to 4th –year students.

Please decide what you would do if you were the ***recipient***.

Possible proposals the other player may make are listed below. Please decide whether you would accept or reject each and circle the appropriate response.

	If the other player proposed the following, what would you do?	**Your decision** (Circle one)	
1	To other player ¥1000, **To you ¥0**	Accept	Reject
2	To other player ¥900, **To you ¥100**	Accept	Reject
3	To other player ¥800, **To you ¥200**	Accept	Reject
4	To other player ¥700, **To you ¥300**	Accept	Reject
5	To other player ¥600, **To you ¥400**	Accept	Reject
6	To other player ¥500, **To you ¥500**	Accept	Reject
7	To other player ¥400, **To you ¥600**	Accept	Reject
8	To other player ¥300, **To you ¥700**	Accept	Reject
9	To other player ¥200, **To you ¥800**	Accept	Reject
10	To other player ¥100, **To you ¥900**	Accept	Reject
11	To other player ¥0, **To you ¥1000**	Accept	Reject

Please ensure you have not missed any responses and close your booklet.

Take care that others cannot see your work.

Enter your ID number here.

↓

#4-2
First year game decision sheet for 2nd to 4th year students

For 2nd to 4th –year students

Decision sheets: Third transaction

The third transaction will now begin.

- In this transaction the other player **will be someone different than in the previous transaction**.
- Do not turn the page until instructed to do so by the experimenter.
- During the experiment **it is strictly forbidden to try to look at the work of those around you**.

Please wait until everyone is ready to begin.

Do not turn the page until everyone is instructed to do so by the experimenter.

You will be paired with
one of the 1st –year students.

Please decide what you would do
if you were the ***proposer***.

For this monetary transaction you have been given ¥1000. How would you like to divide it with the other player? Please write your proposal in the following box.

Of ¥1000:	
Yourself	¥ ______
Other player	¥ ______
Total	¥ 1000

Please wait until everyone has finished.
However, please take care others cannot see your proposal.

You will be paired with one of the 1st –year students.

Please decide what you would do if you were the ***recipient***.

Possible proposals the other player may make are listed below. Please decide whether you would accept or reject each and circle the appropriate response.

	If the other player proposed the following, what would you do?	**Your decision** (Circle one)	
1	To other player ¥1000, **To you ¥0**	Accept	Reject
2	To other player ¥900, **To you ¥100**	Accept	Reject
3	To other player ¥800, **To you ¥200**	Accept	Reject
4	To other player ¥700, **To you ¥300**	Accept	Reject
5	To other player ¥600, **To you ¥400**	Accept	Reject
6	To other player ¥500, **To you ¥500**	Accept	Reject
7	To other player ¥400, **To you ¥600**	Accept	Reject
8	To other player ¥300, **To you ¥700**	Accept	Reject
9	To other player ¥200, **To you ¥800**	Accept	Reject
10	To other player ¥100, **To you ¥900**	Accept	Reject
11	To other player ¥0, **To you ¥1000**	Accept	Reject

Please ensure you have not missed any responses and close your booklet.

Take care that others cannot see your work.

Enter your ID number here.

⬇

#5
Peer-to-peer game decision sheet for all participants

Decision sheets: Fourth transaction

The fourth transaction will now begin.

- In this transaction the other player **will be someone different than in the previous transaction**.
- Do not turn the page until instructed to do so by the experimenter.
- During the experiment **it is strictly forbidden to try to look at the work of those around you**.

Please wait until everyone is ready to begin.

Do not turn the page until everyone is instructed to do so by the experimenter.

You will be paired with another student from the same school year.

Please decide what you would do
if you were the ***proposer***.

For this monetary transaction you have been given ¥1000. How would you like to divide it with the other player? Please write your proposal in the following box.

Of ¥1000:	
Yourself	¥ ______
Other player	¥ ______
Total	¥ 1000

Please wait until everyone has finished.
However, please take care others cannot see your proposal.

You will be paired with another student from the same school year.

Please decide what you would do if you were the ***recipient***.

Possible proposals the other player may make are listed below. Please decide whether you would accept or reject each and circle the appropriate response.

	If the other player proposed the following, what would you do?	**Your decision** (Circle one)	
1	To other player ¥1000, **To you ¥0**	Accept	Reject
2	To other player ¥900, **To you ¥100**	Accept	Reject
3	To other player ¥800, **To you ¥200**	Accept	Reject
4	To other player ¥700, **To you ¥300**	Accept	Reject
5	To other player ¥600, **To you ¥400**	Accept	Reject
6	To other player ¥500, **To you ¥500**	Accept	Reject
7	To other player ¥400, **To you ¥600**	Accept	Reject
8	To other player ¥300, **To you ¥700**	Accept	Reject
9	To other player ¥200, **To you ¥800**	Accept	Reject
10	To other player ¥100, **To you ¥900**	Accept	Reject
11	To other player ¥0, **To you ¥1000**	Accept	Reject

Please ensure you have not missed any responses and close your booklet.

Take care that others cannot see your work.
